# Vasorelaxant Effect of *Boesenbergia rotunda* and Its Active Ingredients on an Isolated Coronary Artery

**DOI:** 10.3390/plants9121688

**Published:** 2020-12-01

**Authors:** Deepak Adhikari, Dal-Seong Gong, Se Hee Oh, Eun Hee Sung, Seung On Lee, Dong-Wook Kim, Min-Ho Oak, Hyun Jung Kim

**Affiliations:** 1 College of Pharmacy and Natural Medicine Research Institute, Mokpo National University, Muan-gun 58554, Korea; dpak7adh@gmail.com (D.A.); nh4011@naver.com (D.-S.G.); yuhui16@naver.com (S.H.O.); hannn0828@hanmail.net (E.H.S.); 2 Department of Oriental Medicine Resources, Mokpo National University, Muan-gun 58554, Korea; lso6918@naver.com (S.O.L.); dbkim@mokpo.ac.kr (D.-W.K.)

**Keywords:** *Boesenbergia rotunda*, Zingiberaceae, flavonoid, 4-hydroxypanduratin, vasorelaxation

## Abstract

Cardiovascular diseases are a major cause of death in developed countries. The regulation of vascular tone is a major approach to prevent and ameliorate vascular diseases. As part of our ongoing screening for cardioprotective natural compounds, we investigated the vasorelaxant effect of rhizomes from *Boesenbergia rotunda* (L.) Mansf. [*Boesenbergia pandurata* (Roxb.) Schltr.] used as a spice and herbal medicine in Asian countries. The methanol extract of *B. rotunda* rhizomes (BRE) exhibited significant vasorelaxation effects ex vivo at EC_50_ values of 13.4 ± 6.1 μg/mL and 40.9 ± 7.9 μg/mL, respectively, with and without endothelium in the porcine coronary artery ring. The intrinsic mechanism was evaluated by treating with specific inhibitors or activators that typically affect vascular reactivity. The results suggested that BRE induced relaxation in the coronary artery rings via an endothelium-dependent pathway involving NO-cGMP, and also via an endothelium-independent pathway involving the blockade of Ca^2+^ channels. Vasorelaxant principles in BRE were identified by subsequent chromatographic methods, which revealed that flavonoids regulate vasorelaxant activity in BRE. One of the flavonoids was a Diels-Alder type adduct, 4-hydroxypanduratin A, which showed the most potent vasorelaxant effect on porcine coronary artery with an EC_50_ of 17.8 ± 2.5 μM. Our results suggest that rhizomes of *B. rotunda* might be of interest as herbal medicine against cardiovascular diseases.

## 1. Introduction

Cardiovascular diseases are a major cause of death in developed countries. The vascular tone plays an important role in the regulation of blood pressure. High blood pressure is a major risk factor for cardiovascular diseases such as hyperpiesia, arrhythmia, heart failure, and atherosclerosis. Therefore, the regulation of vascular tone is one of the major approaches designed to prevent and ameliorate vascular diseases [1]. Several epidemiological studies have suggested that diets rich in polyphenols including fruits and vegetables and beverages protect against cardiovascular risks [2,3,4,5]. As part of our ongoing screening for cardioprotective phytochemicals, the extract from *Boesenbergia rotunda* rhizomes was found to exhibit significant vasorelaxation effects in the coronary artery.

*Boesenbergia rotunda* (L.) Mansf. [*Boesenbergia pandurata* (Roxb.) Schltr.; *Kaempferia pandurata* Roxb.], commonly known as fingerroot or Chinese keys, is a perennial culinary herb used as a flavoring food ingredient belonging to the family Zingiberaceae. It is cultivated and produced in India and southeastern Asian countries such as Indonesia, Thailand, Malaysia and Southwest China [6,7]. The underground parts of this plant have been widely used as traditional medicine to treat cough, muscular pain, rheumatism, gout, infections, inflammatory lesions, and gastrointestinal disorders in Southeast Asia [7]. Intensive studies analyzing the phytochemical composition and biological evaluations of *B. rotunda* rhizomes have been investigated based on these applications [7,8]. Phytochemicals including flavanones, flavones, chalcones and prenylated flavonoids were identified as the main secondary metabolites contributing to the potential biological and medicinal effects [7,8]. The extracts and flavonoids derived from *B. rotunda* rhizomes revealed antioxidant [9], anti-inflammatory [10,11], antimicrobial [12,13,14,15,16], antitumor [17,18,19], anti-aging [20,21], skin-whitening [22], antiulcer [23] and anti-obesity [24,25] activities. However, the vasodilatory effect of *B. rotunda* rhizomes has yet to be reported. In the present study, we investigated the vasorelaxant effect of *B. rotunda* rhizome extracts and the underlying mechanism, followed by isolation and structural elucidation of the key vasodilators in the active extract.

## 2. Results and Discussion

### 2.1. Vascular Relaxantion Induced by B. rotunda Rhizomes on Coronary Artery

**Figure 1:**
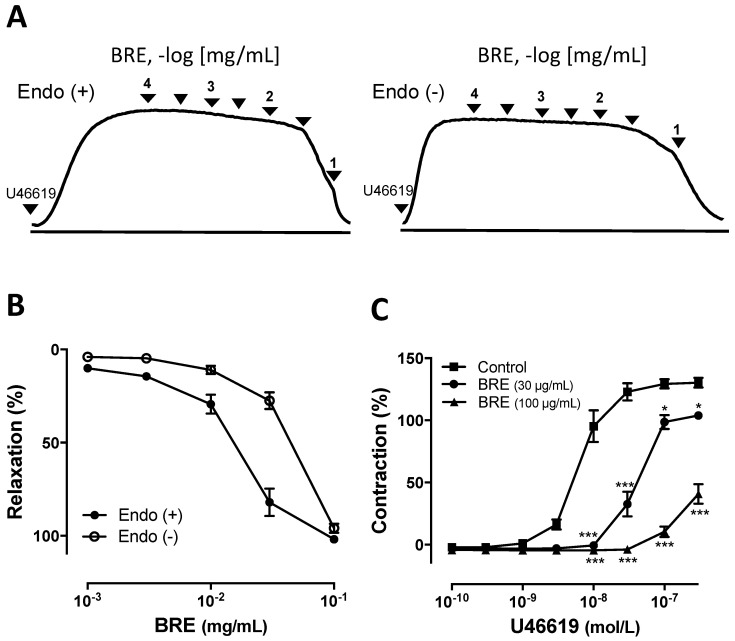
*Boesenbergia rotunda* rhizome methanol extract (BRE) induced concentration-dependent relaxation and prevented contraction in porcine coronary artery rings. Intact and endothelium-denuded rings were contracted with U46619 before the construction of BRE concentration-relaxation. (**A**) representative tracing; (**B**) accumulated figure; (**C**) coronary artery rings were treated with BRE at the indicated concentration before the construction of a concentration–contraction curve in response to U46619. Results are shown as mean ± SEM of 5–10 experiments. * *p* < 0.05, *** *p* < 0.001 versus control.

Dried rhizomes of *B. rotunda* were milled and extracted with MeOH at room temperature, and the filtered liquid extract was evaporated in vacuo to yield a crude extract. The effect of *B. rotuda* rhizome methanol extract (BRE) on the vascular tone was assessed using porcine coronary artery rings contracted submaximal with the thromboxane A_2_ analogue, U46619. BRE induced concentration-dependent relaxation in the endothelial rings, with an EC_50_ value of 13.4 ± 6.1 μg/mL (Figure 1A,B). The endothelium-dependent relaxation was initiated at concentrations greater than 3 μg/mL and reached a near maximal value at 100 μg/mL (E_max_ = 101.8 ± 4.2%, Figure 1A,B). Next, the possibility that BRE, besides inducing vasorelaxations, also affects the contractile responses was assessed. Both 30 and 100 μg/mL of BRE also significantly inhibited the concentration-dependent contractile response to U46619 in a nonparallel fashion and suppressed their maximal responses to 103.9 ± 3.2% and 40.7 ± 10.3% respectively (versus control group 130.3 ± 5.6% compared with 80 mM KCl) in endothelium-intact artery rings (Figure 1C).

To elucidate the role of endothelium in BRE-mediated relaxation in the porcine coronary artery, the concentration responses to BRE were evaluated in endothelium-denuded rings pre-contracted by U46619. BRE caused endothelium-independent relaxation of coronary artery, with an EC_50_ of 40.9 ± 7.9 μg/mL and E_max_ of 97.8 ± 5.3% at 100 μg/mL (Figure 1A,B). The EC_50_ value of vasorelaxant BRE in endothelium-intact coronary artery pre-contracted using U46619 was significantly lower compared with that of endothelium-denuded coronary artery, suggesting that vasorelaxant effects of BRE were mediated in both an endothelium-dependent and -independent manner.

### 2.2. Mechansim of BRE-Induced Vasorelaxation

**Figure 2:**
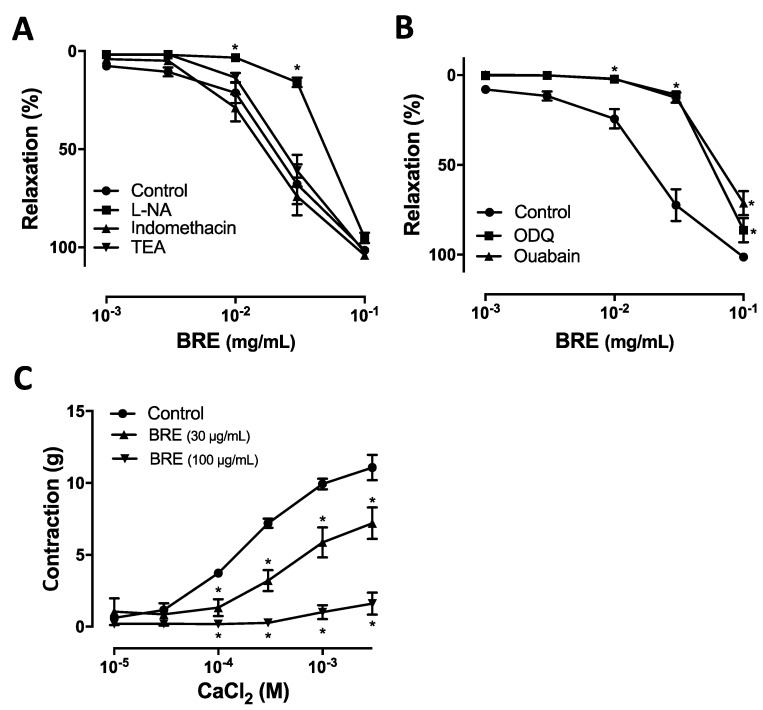
Cumulative concentration–response curves of BRE in coronary artery rings precontracted with U46619 in the presence of (**A**) L-NA (nitric oxide synthase inhibitor, 100 μM), indomethacin (cyclooxygenase inhibitor, 10 μM), TEA (non-selective potassium channel blocker) and (**B**) ODQ (guanylyl cyclase inhibitor, 10 μM), and ouabain (Na^+^/K^+^-ATPase inhibitor, 1 μM). (**C**) BRE inhibits vasoconstriction induced by calcium. Coronary artery rings were treated with BRE at the indicated concentration before drawing a concentration–contraction curve in response to CaCl_2_. Results are shown as mean ± SEM of 5–10 experiments. * *p* < 0.05 versus control.

Vascular endothelium is a barrier separating circulating blood from vascular smooth muscle, and is considered an important dynamic organ regulating vascular tone by releasing various vasoactive factors such as vasodilatory factors [nitric oxide (NO), prostacyclin (PGI_2_) and endothelium derived hyperpolarizing factors (EDHF) etc.] and vasoconstricting factors [thromboxane (TBXs) and endothelin etc.] [26]. Pre-incubation of endothelial rings with L-nitroarginine (L-NA, 100 μM), an inhibitor of nitric oxide synthase (NOS), significantly reduced the BRE-induced relaxation, with an EC_50_ value of 52.2 ± 12.2 μg/mL (versus 13.4 ± 6.1 μg/mL in the absence of L-NA, Figure 1B and Figure 2A). However, indomethacin, a cyclooxygenase inhibitor did not significantly affect the BRE-induced relaxation in endothelium-intact rings (Figure 2A). It was previously reported that the release of NO-induced vasodilation via activation of soluble guanylate cyclase [27] and stimulation of Na^+^/K^+^-ATPase in aortic rings [28]. Therefore, we tested whether or not the vasorelaxant effect of BRE was affected by 1*H*-[1,2,4]oxadiazole[4,3-alpha]quinoxalin-1-one (ODQ, 10 μM), an inhibitor of guanylate cyclase, and ouabain (1 μM), an inhibitor of Na^+^/K^+^-ATPase. ODQ and ouabain significantly reduced BRE-induced relaxation, with EC_50_ values of 64.3 ± 10.1 μg/mL and 80.5 ± 13.4 μg/mL, respectively (versus 13.4 ± 6.1 μg/mL without inhibitors, Figure 2B). These results suggest that the activation of eNOS may play a major role, at least in part, in BRE-induced vasorelaxation.

However, the observation that the vasorelaxant effect of BRE persisted in endothelium-denuded coronary artery ring or in those treated with L-NA suggests that BRE has a direct effect on vascular smooth muscle cells. The opening of K^+^ channels in the vascular smooth muscle cells induces membrane potential hyperpolarization, which is an important mechanism of arterial dilation [29]. However, tetraethylammonium (TEA, 1 mM) which is a K^+^ channel blocker, did not significantly affect the BRE-induced relaxation in the artery ring, suggesting that the vasodilating effect of BRE was not associated with the K^+^ channel (Figure 2A). Another important vascular smooth muscle contraction is mediated by Ca^2+^ influx. BRE significantly and dose-dependently stimulated CaCl_2_-induced contraction in endothelium-denuded coronary artery rings in Ca^2+^-free medium containing 60 mM KCl. The maximal contraction induced by CaCl_2_ (3 mM) was 11.1 ± 0.7 g, 7.2 ± 1.8 g, and 1.6 ± 0.5 g in the absence and presence of BRE (30 μg/mL and 100 μg/mL), respectively (Figure 2C). Taken together, our results suggest that BRE induces relaxation in coronary artery rings via an endothelium-dependent pathway, involving NO-cGMP, and also via an endothelium-independent pathway, involving blockade of Ca^2+^ channels.

### 2.3. Isolation and Structural Elucidaton of Flavonoids in the Rhizomes of B. rotunda

The BRE inducing vasorelaxation was fractionated via liquid–liquid partition based on solvent polarity to afford ethyl acetate (EtOAc), *n*-butyl alcohol (BuOH) and aqueous residue. The EC_50_ values were 15.5 ± 5.2 μg/mL and more than 100 μg/mL for EtOAc and aqueous fractions, respectively. Vascular reactivity showed that the EtOAc fraction exhibited similar vasorelaxant activity compared with that of the crude BRE (13.4 ± 6.1 μg/mL). The BuOH fraction also showed vasorelaxation effects (EC_50_ value, 35.3 ± 12.1 μg/mL), which were weaker than that of the EtOAc fraction, and residual aqueous layer was not active at the highest concentration tested (100 μg/mL). Interestingly, HPLC profiles of active fractions prepared with EtOAc and BuOH revealed the presence of flavonoids in UV spectral data of PDA, while no peaks corresponding to flavonoids were detected in the chromatogram of aqueous residue obtained under the same LC condition (Figure S1).

**Figure 3:**
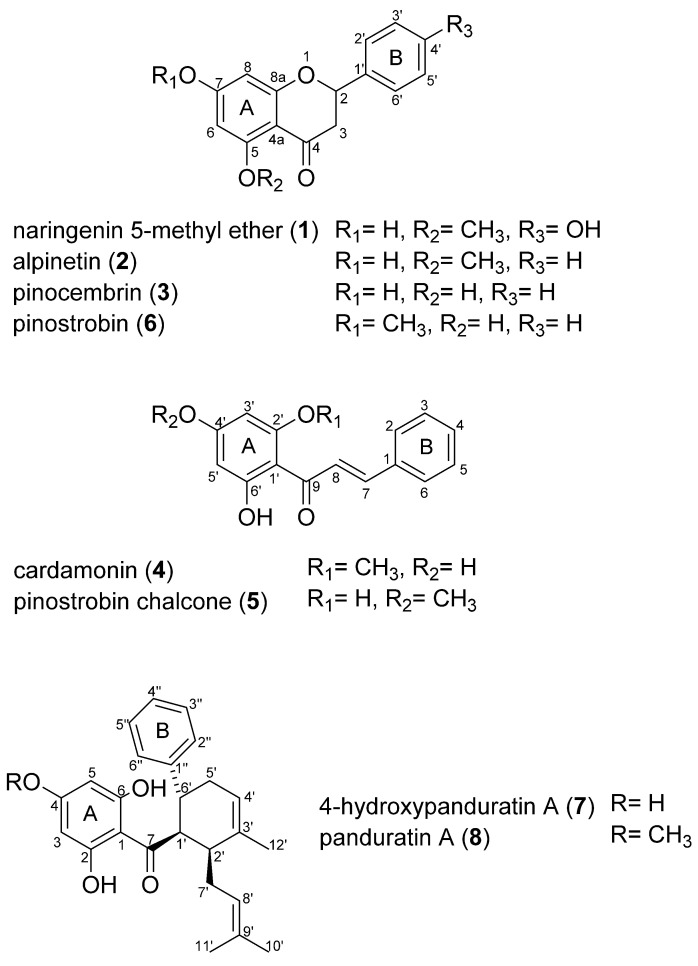
Structures of compounds isolated from *B. rotunda* rhizomes.

**Figure 4:**
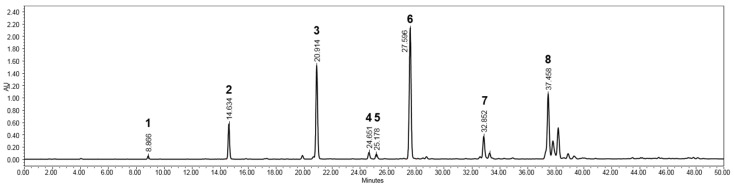
HPLC profile of the BRE derived from *B. rotunda* rhizomes. The chromatogram was based on the following conditions: a linear gradient solvent system of acetonitrile and 0.1% HCOOH-containing water, ranging from 20% to 90% acetonitrile, for 40 min, followed by isocratic elution with 100% acetonitrile for 10 min, on a SunFire C18 (5 μm, 4.6 × 150 mm) waters column, at a flow rate of 1.0 mL/min, and detection under 300 nm; naringenin 5-methyl ether (**1**), alpinetin (**2**), pinocembrin (**3**), cardamonin (**4**), pinostrobin chalcone (**5**), pinostrobin (**6**), 4-hydroxypanduratin A (**7**) and panduratin A (**8**).

The EtOAc fraction was chromatographed by MPLC, and further purified using preparative HPLC to afford eight flavonoids (**1**–**8**) (Figure 3). Their structures were identified by spectroscopic analysis including NMR, UV, and MS, and compared with previously reported spectral data [30,31,32,33,34,35]. These flavonoids were also detected as major peaks in the HPLC chromatogram of BRE (Figure 4).

Naringenin 5-methyl ether (**1**), alpinetin (**2**), pinocembrin (**3**), and pinostrobin (**6**) exhibited similar UV spectral patterns with maximal absorption between 284 and 288 nm in HPLC-PDA analysis, which suggested that their structures carried a flavonoid subgroup, flavanone. The ^1^H NMR data of all these compounds revealed the presence of a 2-phenyl-4-chromanone skeleton including two aromatic ring protons (δ 5.94–6.15, d, H-6 and H-8) with typical meta-coupling constants (1.2–2.4 Hz), an oxymethine proton signal (δ 5.32–5.63, dd, H-2) with *J* values (12–13 and 3.0 Hz), and two methylene proton signals (δ 2.51–2.82 and δ 2.98–3.31, dd, H-3) with coupling constants of 16–17, 12–13 Hz and 16–17, 3.0 Hz, respectively. Their ^13^C NMR data also displayed a 4-chromanone moiety including one carbonyl carbon (C-4), two alicyclic carbons (C-2 and C-3), and six aromatic carbons (C-5~C-8, C-4a, C-8a) as well as a phenyl moiety with six aromatic carbons (C-1′–C-6′). The aromatic protons of the ring B in **2**, **3** and **6** included five protons at δ 7.36–7.57 (H-2′–H-6′) indicating an unsubstituted B-ring, while phenyl protons in naringenin 5-methyl ether (**1**) showed a set of AA’BB’ protons as two doublets (each 2H) at δ 6.77 (C-3′,5′) and δ 7.28 (C-2′,6′) indicating para-substituted ring B. The position of each methoxy group in **1**, **2**, and **6** was identified based on long range couplings in HMBC spectra, respectively.

Cardamonin (**4**) and pinostrobin chalcone (**5**) displayed UV spectra of chalcones with essential and maximal absorption band at nearby 340 nm via HPLC-PDA analysis. NMR data of these compounds revealed that the presence of an α,β-unsaturated ketone with two aromatic rings. The NMR data of both compounds showed two trans-olefin protons (δ 8.02/8.30 and δ 7.75/7.79, H-7 and H-8, d) with a large coupling constant (15.6 Hz) correlating with one carbonyl carbon (δ 193.2 and δ 194.2, C-9). Additionally, their ^1^H NMR spectra showed an unsubstituted phenyl ring possessing five aromatic protons at δ 7.43–7.73 (H-2–H-6), and a 1,2,4,6-tetrasubstituted benzene ring including two aromatic meta-protons at around δ 6.00 (H-3′ and H-5′). The positions of methoxy functions were respectively identified at C-2′ and C-4′ in **4** and **5** by HSQC and HMBC spectra.

4-Hydroxypanduratin A (**7**) and panduratin A (**8**) showed closely similar UV spectra with the maximal absorptions at around UV 290 nm suggesting a conjugated ketone moiety and revealed the difference in 14 mass units between two compounds. The ^1^H NMR data of both shared five aromatic protons of a phenyl group at δ 7.05–7.22 (H-2″–C-6″) and two protons in a 2,4,6-trihydroxyphenyl group near δ 5.90 (2H, H-3,5). They also revealed the presence of a dimethylallyl group consisting of two proton signals in a methylene bridge (δ 2.08–2.29, H-7′), an olefinic proton signal (δ 4.94/4.88, respectively, H-8′) and singlets of dimethyl protons (δ 1.51–1.52, 6H, H-10′ and 11′), as well as a 3-methyl-cyclohex-3-ene moiety with trans–cis configuration (*J*_1′,6′_ = 10.0–11.7 Hz, *J*_1′,2′_ = 4.5–5.5 Hz) corresponding to five aliphatic proton signals at δ 1.99–4.80 (each proton of H-1′, 2′, 6′ and two proton signals of H-5′), an olefinic signal at δ 5.41/5.42 (H-4′) and an allylic methyl at δ1.77 (3H, H-12′). The ^1^H NMR spectrum of panduratin A (**8**) displayed an additional proton signal at δ 3.74 (3H, s). These observations indicated that both compounds were closely related Diels-Alder adducts with a distinct methyl group with long range correlation to the oxygenated aromatic carbon C-4. The presence of all the isolated compounds were previously reported in the rhizomes of *B. rotunda* [8].

### 2.4. Vasorelaxant Activity of Flavonoids from B. rotunda

**Figure 5:**
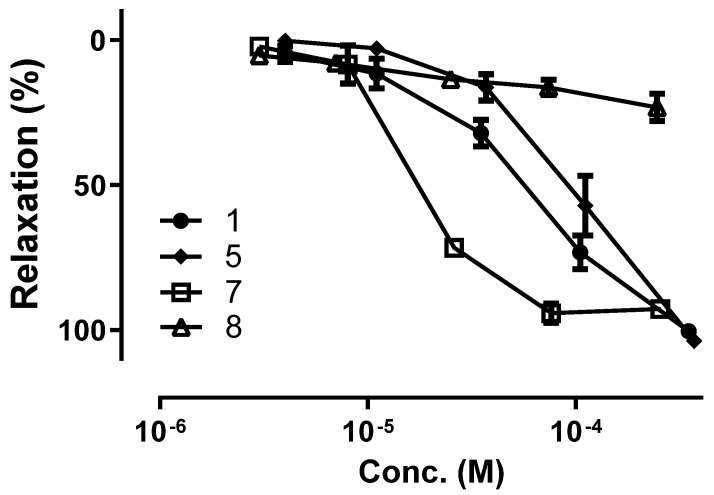
Concentration-response curves illustrating the vasorelaxant effect of naringenin 5-methyl ether (**1**), pinostrobin chalcone (**5**), 4-hydroxypanduratin A (**7**) and panduratin A (**8**). Results are shown as mean ± SEM of 5–10 experiments.

The vasorelaxant effect of the isolated compounds derived from BRE indicated that naringenin 5-methyl ether (**1**), alpinetin (**2**), pinocembrin (**3**), pinostrobin (**6**) and 4-hydroxypanduratin A (**7**) elicited significant vasodilation of coronary artery (Table 1 and Figure 5). Vascular reactivity of flavonoids (**1**–**8**) from BRE described herein suggests a structure-activity relationship underlying for the role and potential of flavonoid scaffold in part. Chalcones including cardamonin (**4**) and pinostrobin chalcone (**5**) showed significantly less potent vasorelaxant effects than those of flavanones including naringenin 5-methyl ether (**1**), alpinetin (**2**), pinocembrin (**3**) and pinostrobin (**6**). Additionally, with functional moieties of the flavanones (**1**–**3**, and **6**), the comparison of EC_50_ values indicated that the vascular reactivity is affected by not only the hydroxyl groups but also the position of a methoxyl in the A and B rings. Furthermore, the presence of hydroxylation of C-4 in ring A demonstrates the pivotal role of vasorelaxant effects induced by the Diels-Alder type adducts such as panduratin A derivatives. Panduratin A (**8**) with a methoxy function at C-4 did not alter the vascular tone, whereas 4-hydroxypanduratin A (**7**) showed the most potent vascular reactivity among the major flavonoids present in the BRE.

The study of the relationship between dietary factors and cardiovascular mortality suggested that the diet-related cardiometabolic deaths are predominantly observed in the population associated with a low intake of vegetables and fruits [36]. Polyphenols naturally exist in plants and plant products, including fruits and vegetables. Currently, more than 8000 phenolic structures are available, including more than 4000 belonging to the flavonoid class, and several hundred flavonoids are present in edible vegetables [37]. The polyphenol structure is characterized by at least a simple phenol core bearing at least one hydroxyl group and classified according to the arrangement of the carbon atoms and their substituents into two main classes. It is largely admitted that flavonoids derived from vegetables and medicinal plants exhibit cardiovascular protective effects [38]. Among eight flavonoids isolated from BRE in the present study, the vasorelaxant effect of alpinetin (**2**) isolated from *Alpinia henryi* K. Schum. [39], pinocembrin (**3**) from propolis [40,41], cardamonin (**4**) from *A. henryi* K. Schum. [39], and pinostrobin (**6**) from *Teloxys graveolens* [42] was reported previously. Consistent with our results, the vasorelaxant effect of alpinetin (**2**), pinocembrin (**3**) and cardamonin (**4**) was shown both in endothelium-dependent, which was associated with nitric oxide, and endothelium-independent, which was associated with the blockade of Ca^2+^ channels [39,40,41,42]. To the best of our knowledge, the present investigation is the first report suggesting that naringenin 5-methyl ether (**1**) and 4-hydroxypanduratin A (**7**) isolated from rhizomes of *B. rotunda* induce vasorelaxation; however, the molecular mechanism of action remains to be clarified, and cardiovascular effect and toxicity of the isolated vasoactive flavonoids need to be further investigated via in vitro and in vivo studies. Taken together, the BRE induced relaxation in coronary artery via endothelium-dependent and independent mechanisms, whose effects are, at least in part, due to constituents including that naringenin 5-methyl ether (**1**), alpinetin (**2**), pinocembrin (**3**), pinostrobin (**6**) and 4-hydroxypanduratin A (**7**). They further suggest that BRE might be of interest as a source of herbal medicine or functional food to prevent the development of cardiovascular diseases.

## 3. Materials and Methods

### 3.1. Plant Material

The dried rhizomes of *B. rotunda* originating in Indonesia were obtained from an herbal company in Suwon, Korea in 2016, and were identified by Prof. Hyun Jung Kim at the Laboratory of Pharmacognosy, College of Pharmacy, Mokpo National University. A voucher specimen was deposited at the herbarium placed in the same institute (No. P2016BRR001).

### 3.2. Reagents and Chemicals

9,11-Dideoxy-9α,11α-methanoepoxy prostaglandin F_2α_ (U46619) was supplied by Cayman Chemical (Ann Arbor, MI, USA), and indomethacin, N-ω-nitro-L-arginine (L-NA), bradykinin, ODQ, 9-(tetrahydro-2-furanyl)-9*H*-purin-6-amine (SQ22563), ouabain by Sigma-Aldrich (St. Louis, MO, USA). All chemicals were of analytical grade. HPLC grade solvents (Fisher Scientific, Hampton, NH, USA) were used for all the HPLC experiments, and extra pure grade solvents were used for extraction.

### 3.3. Extraction and Isolation of the Rhizomes of B. rotunda

Dried rhizomes of *B. rotunda* (600 g) were milled and extracted with MeOH (1 L × 4) at room temperature, and the filtered liquid extract was evaporated in vacuo to afford a crude extract (91.9 g). An aliquot of this extract (48.7 g) was suspended in 1 L of H_2_O, subsequently partitioned with EtOAc (1 L × 4) and *n*-BuOH (1 L × 3). The EtOAc fraction (25 g) was subjected to Si gel MPLC (Biotage Isolera One, Biotage, Uppsala, Sweden) with Biotage SNAP cartridge KP-SIL 340 g using *n*-hexane and CHCl_3_/acetone (1:1) mixtures at 60 mL/min for 60 min to obtain 20 fractions (F01-F20). The combined F03 through F05 (4.88 g) was purified by preparative HPLC (Waters 600 system, Waters, Milford, MA) with a SunFire^TM^ Prep OBD^TM^ column (5 μm, 19 × 150 mm, Waters) using acetonitrile and 0.1% HCOOH containing water (30:70 to 100% acetonitrile, 30 min) at the flow rate of 10.0 mL/min to give pinostrobin (**6**, 944.0 mg). The fractions F06 and F07 were combined, and an aliquot of a fraction (7 g) was chromatographed with Biotage SNAP Cartridge (KP-C18-HS 120 g) using a gradient solvent system of acetonitrile and water (30:70 to 100% acetonitrile) at 30 mL/min for 60 min to yield eight subfractions (SF01-SF08). Alpinetin (**2**, 94.0 mg) from SF01, pinocembrin (**3**, 69.8 mg) and cardamonin (**4**, 49.4 mg) from SF02, pinostrobin chalcone (**5**, 12.7 mg) from SF03, panduratin A (**8**, 116. 4 mg) from SF05, and 4-hydroxypanduratin A (**7**, 32.9 mg) from SF06, were obtained respectively via preparative HPLC purification with a SunFire^TM^ Prep OBD^TM^ column (5 μm, 19 × 150 mm, Waters) using acetonitrile and 0.1% HCOOH containing water (30:70 to 100% acetonitrile, 30 min) at 10.0 mL/min. F07 was further separated using the same preparative HPLC condition using acetonitrile and 0.1% HCOOH containing water (25:75 to 70:30, 15 min) at the flow rate of 10.0 mL/min to obtain naringenin 5-methyl ether (**1**, 84.0 mg). The structures of all the isolated compounds (**1**–**8**) were elucidated by spectroscopic analysis and compared with previously reported spectral data (Figures S2–S9) [30,31,32,33,34,35].

Naringenin 5-methyl ether (**1**) *m*/*z* 287.1 [M + H]^+^; UV λ_max_ 284 nm; ^1^H NMR (600 MHz, DMSO-*d*_6_): 7.28 (2H, d, *J* = 8.4 Hz, H-2′,6′), 6.77 (2H, d, *J* = 8.4 Hz, H-3′,5′), 6.04 (1H, d, *J* = 1.2 Hz, H-6), 5.94 (1H, d, *J* = 1.2 Hz, H-8), 5.32 (1H, dd, *J* = 12.6, 3.0 Hz, H-2), 3.72 (3H, s, 5-OCH_3_), 2.98 (1H, dd, *J* = 16.2, 12.6 Hz, H-3), 2.51 (1H, dd, *J* = 16.2, 3.0 Hz, H-3); ^13^C NMR (150 MHz, DMSO-*d*_6_): 187.7 (C-4), 164.8 (C-8a), 164.2 (C-7), 162.2 (C-5), 157.6 (C-4′), 129.4 (C-1′), 128.1 (C-2′,6′), 115.1 (C-3′,5′), 104.2 (C-4a), 95.7 (C-8), 93. 4 (C-6), 78.0 (C-2), 55.6 (5-OCH_3_), 44.8 (C-3).

Alpinetin (**2**) *m*/*z* 271.1 [M + H]^+^; UV λ_max_ 286 nm; ^1^H NMR (600 MHz, DMSO-*d*_6_): 7.49 (2H, brd, *J* = 7.3 Hz, H-2′,6′), 7.41 (2H, brt, *J* = 7.3 Hz, H-3′,5′), 7.36 (1H, brt, *J* = 7.3 Hz, H-4′), 6.07 (1H, d, *J* = 1.8 Hz, H-6), 6.00 (1H, d, *J* = 1.8 Hz, H-8), 5.48 (1H, dd, *J* = 12.3, 3.0 Hz, H-2), 3.74 (3H, s, 5-OCH_3_), 2.98 (1H, dd, *J* = 16.2, 12.3 Hz, H-3), 2.62 (1H, dd, *J* = 16.2, 3.0 Hz, H-3); ^13^C NMR (150 MHz, DMSO-*d*_6_): 187.3 (C-4), 164.6 (C-7), 164.1 (C-8a), 162.2 (C-5), 139.2 (C-1′), 128.5 (C-3′,5′), 128.3 (C-4′), 126.4 (C-2′,6′), 104.4 (C-4a), 95.7 (C-8), 93. 4 (C-6), 78.0 (C-2), 55.6 (5-OCH_3_), 44.9 (C-3).

Pinocembrin (**3**) *m*/*z* 257.1 [M + H]^+^; UV λ_max_ 288 nm; ^1^H NMR (600 MHz, acetone-*d*_6_): 12.16 (1H, s, 5-OH), 7.57 (2H, brt, *J* = 7.2 Hz, H-2′,6′), 7.45 (2H, td, *J* = 7.2, 1.8 Hz, H-3′,5′), 7.40 (1H, tt, *J* = 7.2, 1.8 Hz, H-4′), 6.00 (1H, d, *J* = 2.4 Hz, H-6), 5.96 (1H, d, *J* = 2.4 Hz, H-8), 5.58 (1H, dd, *J* = 12.9, 3.0 Hz, H-2), 3.17 (1H, dd, *J* = 17.4, 12.9 Hz, H-3), 2.81 (1H, dd, *J* = 17.4, 3.0 Hz, H-3); ^13^C NMR (150 MHz, acetone-*d*_6_): 196.9 (C-4), 167.7 (C-7), 165.4 (C-5), 164.2 (C-8a), 140.1 (C-1′), 129.6 (C-3′,5′), 129.5 (C-4′), 127.4 (C-2′,6′), 103.3 (C-4a), 97.1 (C-8), 96. 0 (C-6), 80.0 (C-2), 43.7 (C-3).

Cardamonin (**4**) *m*/*z* 271.1 [M + H]^+^; UV λ_max_ 344 nm; ^1^H NMR (600 MHz, acetone-*d*_6_): 8.02 (1H, d, *J* = 15.6 Hz, H-7), 7.75 (1H, d, *J* = 15.6 Hz, H-8), 7.73 (2H, brd, *J* = 7.2 Hz, H-2,6), 7.44 (3H, m, H-3,4,5), 6.09 (1H, d, *J* = 2.2 Hz, H-3′), 6.01 (1H, d, *J* = 2.2 Hz, H-5′), 3.98 (3H, s, 2′-OCH_3_); ^13^C NMR (150 MHz, acetone-*d*_6_): 193.2 (C-9), 169.1 (C-6′), 166.3 (C-4′), 164.4 (C-2′), 142.7 (C-8), 136.5 (C-1), 131.0 (C-4), 129.9 (C-3,5), 129.3 (C-2,6), 128.6 (C-7), 106.4 (C-1′), 97.1 (C-5′), 92.4 (C-3′), 56.5 (2’-OCH_3_).

Pinostrobin chalcone (**5**) *m*/*z* 271.1 [M + H]^+^; UV λ_max_ 340 nm; ^1^H NMR (600 MHz, acetone-*d*_6_): 8.30 (1H, d, *J* = 15.6 Hz, H-7), 7.79 (1H, d, *J* = 15.6 Hz, H-8), 7.70 (2H, dd, *J* = 7.8, 1.8 Hz, H-2,6), 7.44 (2H, m, H-3,5), 7.43 (1H, m, H-4), 6.04 (2H, s, H-3′,5′), 3.81 (3H, s, 4′-OCH_3_); ^13^C NMR (150 MHz, acetone-*d*_6_): 194.2 (C-9), 168.0 (C-4′), 166.5 (C-2′,6′), 143.5 (C-8), 137.2 (C-1), 131.6 (C-4), 130.5 (C-3,5), 129.9 (C-2,6), 129.2 (C-7), 107.0 (C-1′), 95.2 (C-3′,5′), 56.5 (4′-OCH_3_).

Pinostrobin (**6**) *m*/*z* 271.1 [M + H]^+^; UV λ_max_ 288 nm; ^1^H NMR (600 MHz, DMSO-*d*_6_): 12.11 (1H, s, 5-OH), 7.53 (2H, brd, *J* = 7.2 Hz, H-2′,6′), 7.44 (2H, brt, *J* = 7.2 Hz, H-3′,5′), 7.39 (1H, tt, *J* = 7.2, 1.2 Hz, H-4’), 6.15 (1H, d, *J* = 1.8 Hz, H-8), 6.10 (1H, d, *J* = 1.8 Hz, H-6), 5.63 (1H, dd, *J* = 13.2, 3.0 Hz, H-2), 3.79 (3H, s, 7-OCH_3_), 3.31 (1H, dd, *J* = 17.2, 13.2 Hz, H-3), 2.82 (1H, dd, *J* = 17.2, 3.0 Hz, H-3); ^13^C NMR (150 MHz, DMSO-*d*_6_): 196.5 (C-4), 167.5 (C-7), 163.2 (C-5), 162.7 (C-8a), 138.5 (C-1′), 128.6 (C-4′), 128.5 (C-3′,5′), 126.7 (C-2′,6′), 102.7 (C-4a), 94.8 (C-6), 93. 9 (C-8), 78.6 (C-2), 55.9 (5-OCH_3_), 42.2 (C-3).

4-Hydroxypanduratin A (**7**) *m*/*z* 393.2 [M + H]^+^; UV λ_max_ 291 nm; ^1^H NMR (600 MHz, acetone-*d*_6_): 7.22 (2H, brd, *J* = 7.8 Hz, H-2″,6″), 7.18 (1H, brt, *J* = 7.8 Hz, H-3″,5″), 7.05 (1H, tt, *J* = 7.6, 1.2 Hz, H-4″), 5.89 (2H, s, H-3, 5), 5.41 (1H, s, H-4′), 4.94 (1H, tq, *J* = 7.2, 1.2 Hz, H-8′), 4.80 (1H, dd, *J* = 11.7, 4.5 Hz, H-1′), 3.43 (1H, td, *J* = 10.8, 6.6 Hz, H-6′), 2.69 (1H, q, *J* = 5.4 Hz, H-2′), 2.36 (1H, m, H-5′), 2.29 (1H, dt, *J* = 15.6, 7.2 Hz, H-7′), 2.08 (1H, m, H-7′), 1.99 (1H, ddq, *J* = 18.0, 4.8, 1.8 Hz, H-5’), 1.77 (3H, d, *J* = 1.2 Hz, H-12′), 1.52 (3H, s, H-10′), 1.51 (3H, d, *J* = 1.2 Hz, H-11′); ^13^C NMR (150 MHz, acetone-*d*_6_): 207.1 (C-7), 165.0 (C-2,4,6), 148.4 (C-1″), 138.0 (C-3′), 131.8 (C-9′), 129.0 (C-3″,5″), 128.1 (C-2″,6″), 126.3 (C-4″), 125.5 (C-8′), 121.8 (C-4′), 106.3 (C-1), 96.0 (C-3,5), 54.5 (C-1′), 43.4 (C-2′), 37.9 (C-6′), 36.9 (C-5′), 29.6 (C-7′), 26.0 (C-11′), 23.1 (C-12′), 18.1 (C-10′).

Panduratin A (**8**) *m*/*z* 407.2 [M + H]^+^; UV λ_max_ 290 nm; ^1^H NMR (500 MHz, CDCl_3_): 7.22 (4H, m, H-2″,3″,5″,6″), 7.09 (1H, m, H-4″), 5.91 (2H, s, H-3, 5), 5.42 (1H, brs, H-4′), 4.88 (1H, brt, *J* = 6.0 Hz, H-8′), 4.72 (1H, dd, *J* = 11.5, 4.5 Hz, H-1′), 3.74 (4-OCH_3_), 3.44 (1H, tdd, *J* = 10.0, 6.0, 1.5 Hz, H-6′), 2.64 (1H, q, *J* = 5.5 Hz, H-2′), 2.39 (1H, m, H-5′), 2.27 (1H, dt, *J* = 15.5, 7.5 Hz, H-7′), 2.08 (1H, m, H-7′), 2.02 (1H, m, H-5′), 1.77 (3H, s, H-12′), 1.52 (6H, s, H-10′,11′); ^13^C NMR (125 MHz, CDCl_3_): 206.6 (C-7), 165.1 (C-2,4,6), 147.1 (C-1″), 137.2 (C-3′), 131.8 (C-9′), 128.3 (C-3″,5″), 127.1 (C-2″,6″), 125.5 (C-4″), 124.3 (C-8′), 121.0 (C-4′), 105.9 (C-1), 94.1 (C-3,5), 55.3 (4-OCH_3_), 53.7 (C-1′), 42.5 (C-2′), 37.1 (C-6′), 35.9 (C-5′), 28.9 (C-7′), 25.7 (C-11′), 22.8 (C-12′), 17.9 (C-10′).

### 3.4. HPLC-PDA and LC-MS Analysis

HPLC analysis of extracts was carried out in a Waters HPLC system (Waters Corporation, Milford, MA) composed of a 1525 binary pump with a column oven, a 2707 autosampler, and a 2998 photodiode array detector (210–400 nm) using a Waters SunFire C18 column (5 μm, 4.6 × 150 mm). Plant extract, fractions, and single compounds were eluted by a linear gradient system using acetonitrile and water (0.1% HCOOH), ranging from 20% A to 90% acetonitrile (for 40 min) followed by an isocratic solvent 100% acetonitrile (10 min) at the flow rate of 1.0 mL/min. UV absorption was monitored under 300 nm. LC-ESI-MS experiments were performed on Agilent 6120 single quadruple MS hyphenated to Agilent 1260 Infinity quaternary LC (Agilent Technologies, Santa Clara, CA, USA) using a Thermo Acclaim Polar Advantage II (2.2 μm, 2.1 × 100 mm) in the positive mode, and developed by a gradient solvent mixture with acetonitrile and water (0.1% HCOOH), 20% to 100% acetonitrile for 25 min at 0.3 mL/min. The MS detection was carried out using electrospray ionization (ESI) with API source and MS spectra were obtained between *m*/*z* 100–1000 in a positive mode.

### 3.5. Vascular Reactivity

Pig hearts were collected from the local slaughterhouse (Mokpo, Korea) and the vascular reactivity was assessed using coronary artery as indicated previously [43]. Briefly, the left anterior descending coronary arteries of porcine heart were dissected, cleaned of connective tissue, and cut into rings (4–5 mm in length) carefully. Then, porcine coronary artery rings were incubated in organ baths containing oxygenated (95% O_2_ and 5% CO_2_) Krebs bicarbonate solution (mmol/L; NaCl, 119; KCl, 4.7; KH_2_PO_4_, 1.18; MgSO_4_, 1.18; CaCl_2_, 1.25; NaHCO_3_, 25; and D-glucose, 11; pH 7.4, 37 °C) to determine the changes in isometric tension. Following equilibration for 90 min under a resting tension of 5 g, the rings were contracted twice with KCl (80 mmol/L). Subsequently, the rings were pre-contracted with the thromboxane mimetic U46619 (1–60 nmol/L) to about 80% of the maximal contraction and the integrity of the endothelium was checked with bradykinin (0.3 μmol/L). After washout and a 30 min equilibration period, the rings were again contracted with U46619 before construction of a concentration–relaxation curve involving extracts and isolated compounds. In some experiments analyzing the role of endothelium-derived vasoactive factors, the rings were exposed to various inhibitors for 30 min before the addition of U46619. The inhibition of contractile response was assessed by exposing the rings to extracts for 30 min before construction of concentration-contraction curve either for U46619 or CaCl_2_ in the presence of 40 mM KCl. The contractile response to CaCl_2_ or U46619 was expressed as the percentage of the maximal contraction induced by KCl (80 mmol/L) in a standard Krebs solution.

### 3.6. Statistical Analysis

All data are expressed as mean ± SEM. Statistical analysis of the data was performed using the Student’s *t*-test or multiway ANOVA followed by Fisher’s protected least significant difference test where appropriate. A value of *p* < 0.05 was considered statistically significant.

## 4. Conclusions

This study demonstrates that *B. rotunda* (L.) Mansf. [*B. pandurata* (Roxb.) Schltr.] extracts induce relaxation in coronary artery rings via, at least in part, an endothelium-dependent pathway, involving NO-cGMP, and also via an endothelium-independent pathway, involving blockade of Ca^2+^ channels. Vasoactive flavonoids were identified from the active fraction, including naringenin 5-methyl ether (**1**), alpinetin (**2**), pinocembrin (**3**), pinostrobin (**6**) and 4-hydroxypanduratin A (**7**), which induced significant vasodilation of coronary artery. Although the exact underlying mechanism of each compounds still remains to be elucidated, our findings suggest that rhizomes of *B. rotunda* might be of interest as an herbal medicine and functional food to prevent the development of cardiovascular diseases.

## Figures and Tables

**Table 1:** Values of half maximal effective dose (EC_50_) and the maximum effect (E_max_) of isolated compounds from BRE.

Compound	EC_50_ (μM)	E_max_ (%)
Naringenin 5-methyl ether (**1**)	52.3 ± 10.4	100.4 ± 1.9
Alpinetin (**2**)	85.6 ± 23.9	104.6 ± 8.9
Pinocembrin (**3**)	35.1 ± 15.3	107.2 ± 3.5
Cardamonin (**4**)	166.7 ± 35.5	65.4 ± 7.4
Pinostrobin chalcone (**5**)	92.1 ± 22.7	103.7 ± 3.7
Pinostrobin (**6**)	52.0 ± 14.3	89.9 ± 2.1
4-Hydroxypanduratin A (**7**)	17.8 ± 2.5	92.7 ± 6.8
Panduratin A (**8**)	EC_50_ ≥ 1000	23.1 ± 4.5

## Supplementary Materials

The following are available online at https://www.mdpi.com/2223-7747/9/12/1688/s1, Figure S1. Comparison of HPLC profiles for three fractions derived from the vasoactive MeOH extract of *Boesenbergia rotunda* rhizomes (BRE), Figure S2. ^1^H and ^13^C NMR data of naringenin 5-methyl ether (**1**), Figure S3. ^1^H and ^13^C NMR data of alpinetin (**2**), Figure S4. ^1^H and ^13^C NMR data of pinocembrin (**3**), Figure S5. ^1^H and ^13^C NMR data of cardamonin (**4**), Figure S6. ^1^H and ^13^C NMR data of pinostrobin chalcone (**5**), Figure S7. ^1^H and ^13^C NMR data of pinostrobin (**6**), Figure S8. ^1^H and ^13^C NMR data of 4-hydroxypanduratin A (**7**), Figure S9. ^1^H and ^13^C NMR data of panduratin A (**8**).

## Abbreviations

BRE	*Boesenbergia rotunda* rhizomes methanol extract
HPLC-PDA	High performance liquid chromatography-photodiode array detector
LC-MS	Liquid chromatography-mass spectrometry
eNOS	Endothelial nitric oxide synthase
ODQ	1*H*- [1,2,4]oxadiazole [4,3-alpha]quinoxalin-1-one
L-NA	L-Nitroarginine
TEA	Tetraethylammonium
cGMP	Cyclic guanosine monophosphate
