# Supplementary material for: Vasorelaxant Effect of Boesenbergia rotunda and Its Active Ingredients on an Isolated Coronary Artery
Source: Plants (Basel). 2020 Dec 1;9(12):1688. doi: 10.3390/plants9121688 (PMC7760037; doi:10.3390/plants9121688)
Supplement: Supplementary file 1 [file plants-09-01688-s001.pdf]

Vasorelaxant Effect of *Boesenbergia rotunda* and its Active Ingredients on an Isolated Coronary Artery

Deepak Adhikari [1,#], Dal-Seong Gong [1,#], Se Hee Oh [1], Eun Hee Sung [1], Seung On Lee [2], Dong-Wook Kim [2], Min-Ho Oak [1,*] and Hyun Jung Kim [1,*]

[1] College of Pharmacy and Natural Medicine Research Institute, Mokpo National University, Muan-gun, Jeonnam 58554, Republic of Korea; dpak7adh@gmail.com (D.A.); nh4011@naver.com (D.S.G.); yuhui16@naver.com (S.H.O.); hannn0828@hanmail.net (E.H.S.)

[2] Department of Oriental Medicine Resources, Mokpo National University, Muan-gun, 58554, Republic of Korea; lso6918@naver.com (S.O.L.); dbkim@mokpo.ac.kr (D.W.K.)

[*] Correspondence: mhoak@mokpo.ac.kr (M.H.O); hyunkim@mokpo.ac.kr (H.J.K); Tel.: +82-61-450-2681 (M.H.O.); Tel.: +82-61-450-2686 (H.J.K.)

[#] The authors contributed to this work equally.

Contents

Figure S1. Comparison of HPLC profiles for three fractions derived from the vasoactive MeOH extract of *Boesenbergia rotunda* rhizomes (BRE).

Figure S2. ^{1}H NMR (600 MHz) and ^{13}C NMR (150 MHz) data of naringenin 5-methyl ether (**1**)

Figure S3. ^{1}H NMR (600 MHz) and ^{13}C NMR (150 MHz) data of alpinetin (**2**)

Figure S4. ^{1}H NMR (600 MHz) and ^{13}C NMR (150 MHz) data of pinocembrin (**3**)

Figure S5. ^{1}H NMR (600 MHz) and ^{13}C NMR (150 MHz) data of cardamonin (**4**)

Figure S6. ^{1}H NMR (600 MHz) and ^{13}C NMR (150 MHz) data of pinostrobin chalcone (**5**)

Figure S7. ^{1}H NMR (600 MHz) and ^{13}C NMR (150 MHz) data of pinostrobin (**6**)

Figure S8. ^{1}H NMR (600 MHz) and ^{13}C NMR (150 MHz) data of 4-hydroxypanduratin A (**7**)

Figure S9. ^{1}H NMR (500 MHz) and ^{13}C NMR (125 MHz) data of panduratin A (**8**)

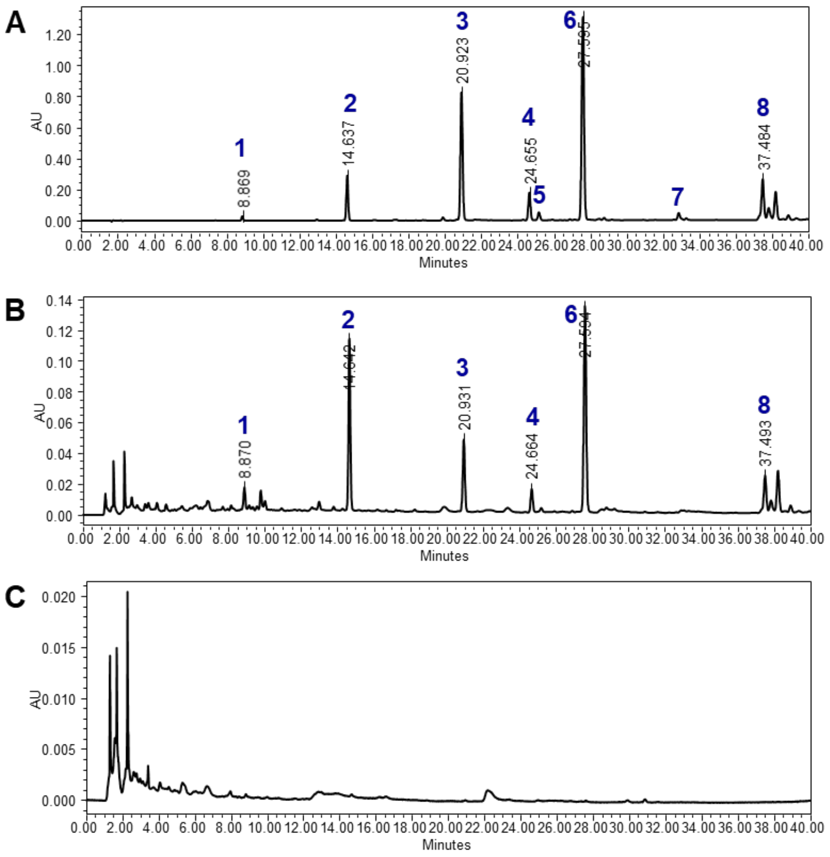

Figure S1. Comparison of HPLC profiles for three fractions derived from the vasoactive MeOH extract of *Boesenbergia rotunda* rhizomes (BRE). The chromatograms of EtOAc (A), *n*-BuOH (B) and aqueous layer (C) were reported in the following condition; a linear gradient solvent system of acetonitrile and 0.1% HCOOH-containing water, ranging from 20% to 90% acetonitrile, for 40 min followed by an isocratic elution with 100% acetonitrile for 10 min, SunFire C18 (5 µm, 4.6 × 150 mm, Waters) column, 1.0 mL/min of flow rate, and detection under 300 nm; naringenin 5-methyl ether (**1**), alpinetin (**2**), pinocembrin (**3**), cardamonin (**4**), pinostrobin chalcone (**5**), pinostrobin (**6**), 4-hydroxypanduratin A (**7**) and panduratin A (**8**).

Figure S2. ^{1}H and ^{13}C NMR data of naringenin 5-methyl ether (**1**)

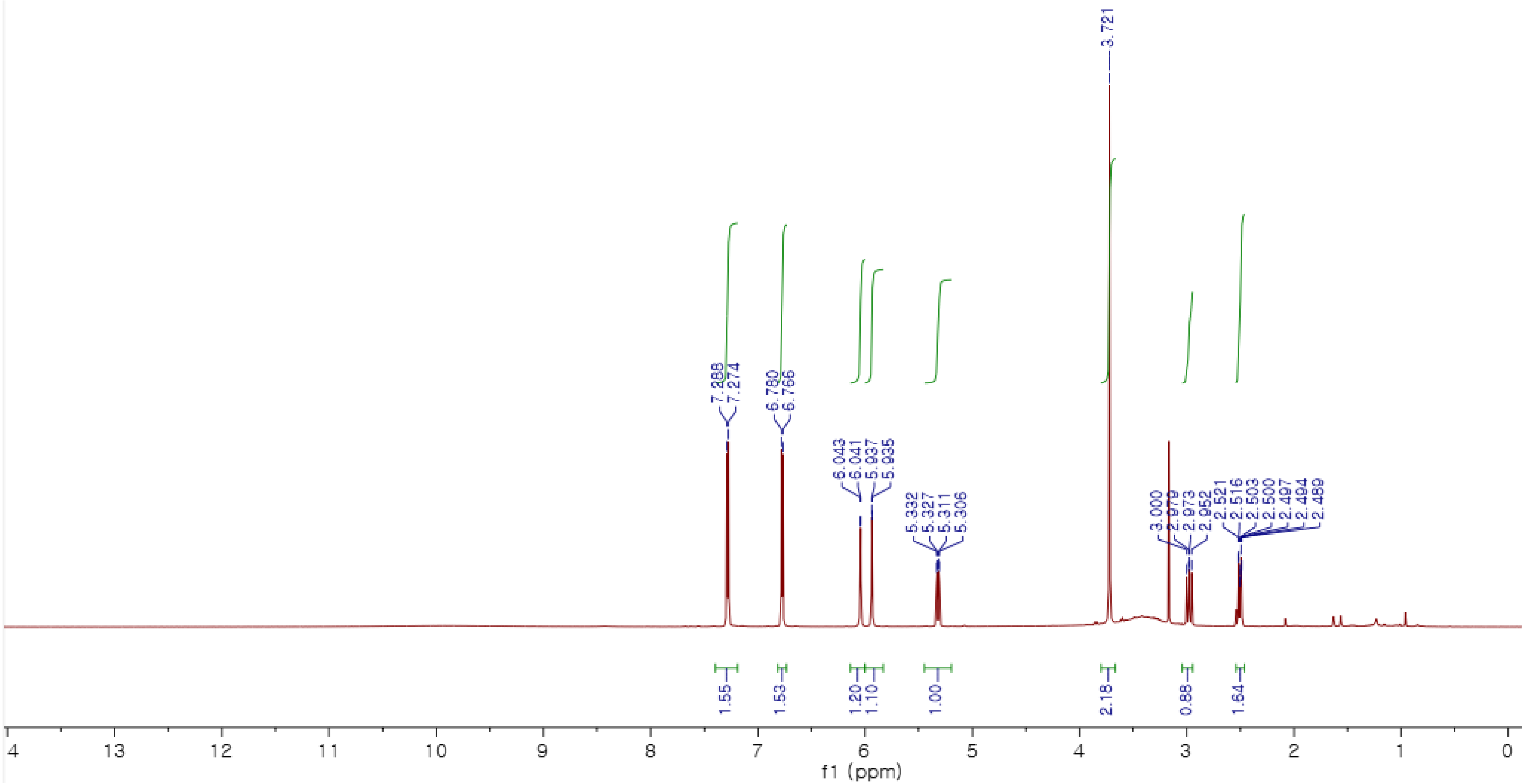

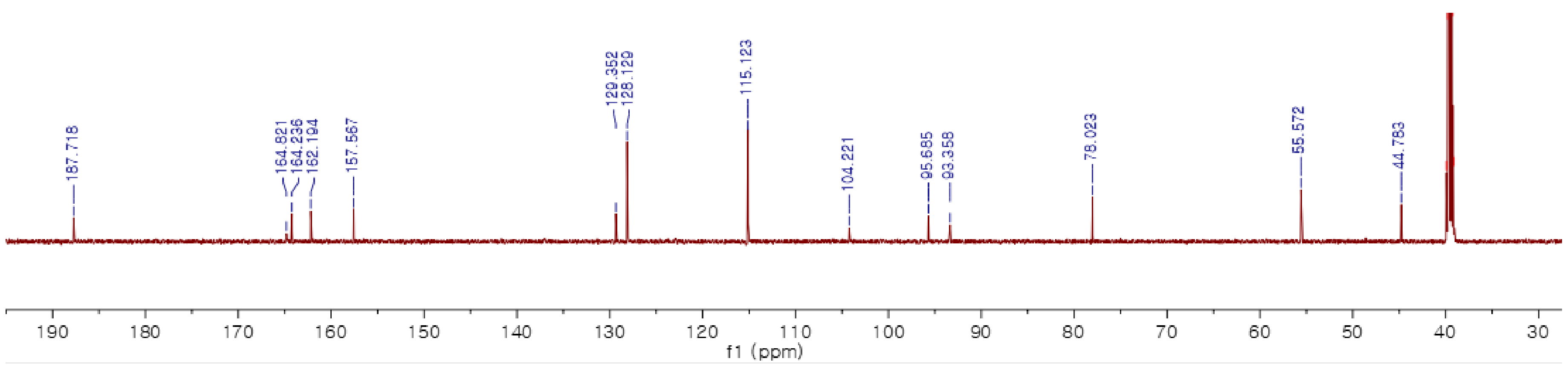
187.718
164.821
164.236
162.194
157.567
129.352
128.129
115.123
104.221
95.685
93.358
78.023
55.572
44.783
190 180 170 160 150 140 130 120 110 100 90 80 70 60 50 40 30
f1 (ppm)

Figure S3. ^{1}H and ^{13}C NMR data of alpinetin (**2**)

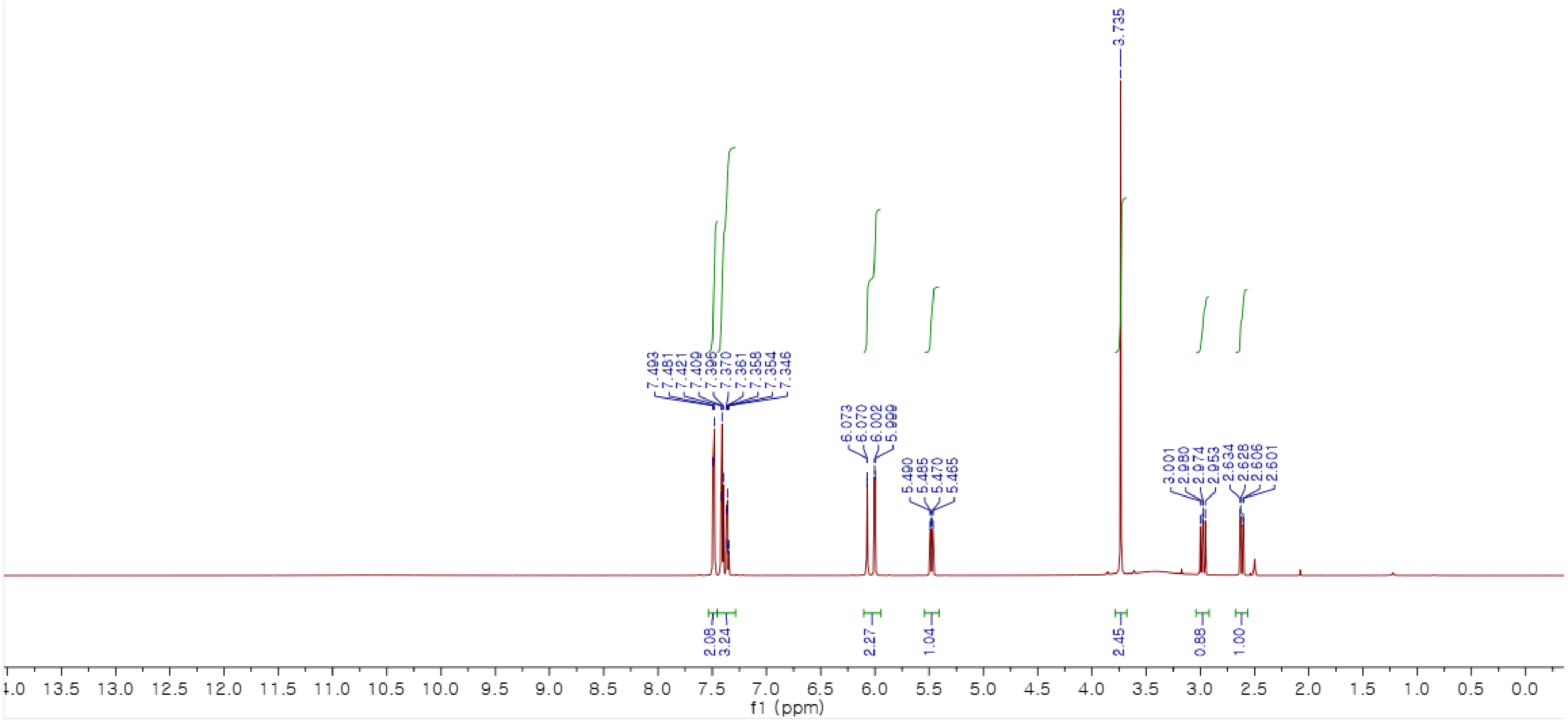

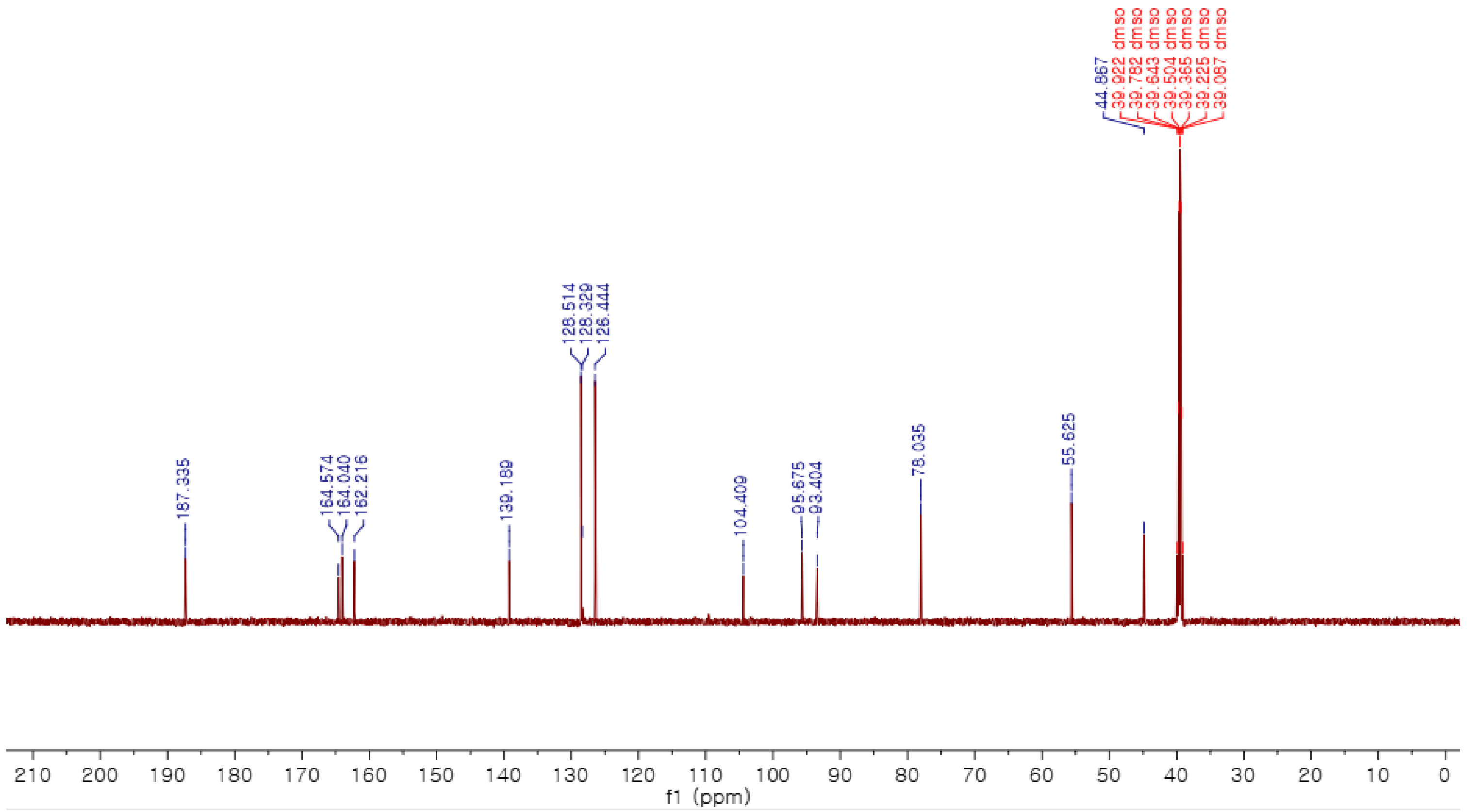
187.335
164.574
164.040
162.216
139.189
128.514
128.329
126.444
104.409
95.675
93.404
78.035
55.625
44.867
39.922 dmso
39.782 dmso
39.643 dmso
39.504 dmso
39.365 dmso
39.225 dmso
39.087 dmso
210 200 190 180 170 160 150 140 130 120 110 100 90 80 70 60 50 40 30 20 10 0
f1 (ppm)

Figure S4. ^{1}H and ^{13}C NMR data of pinocembrin (**3**)

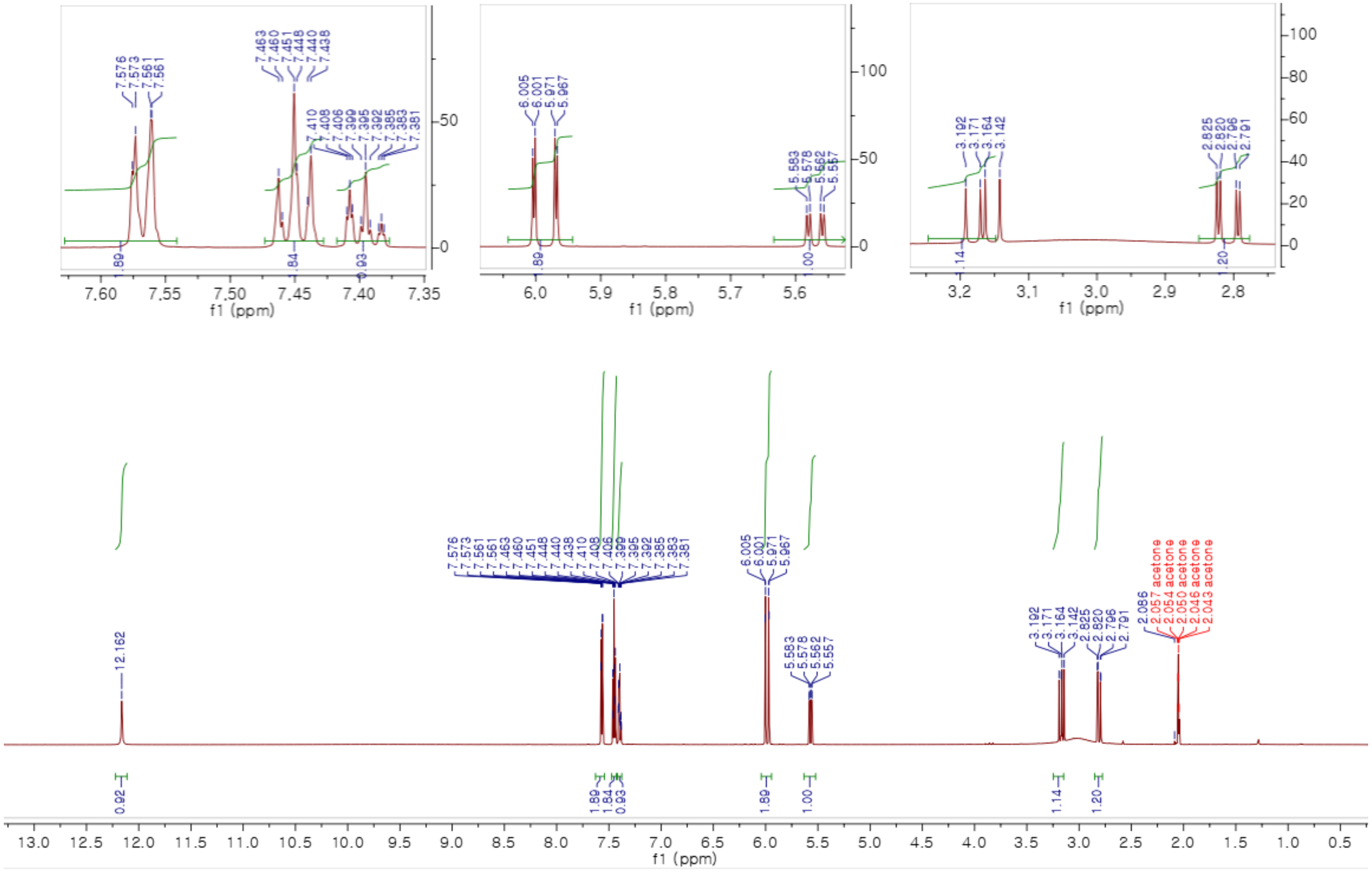

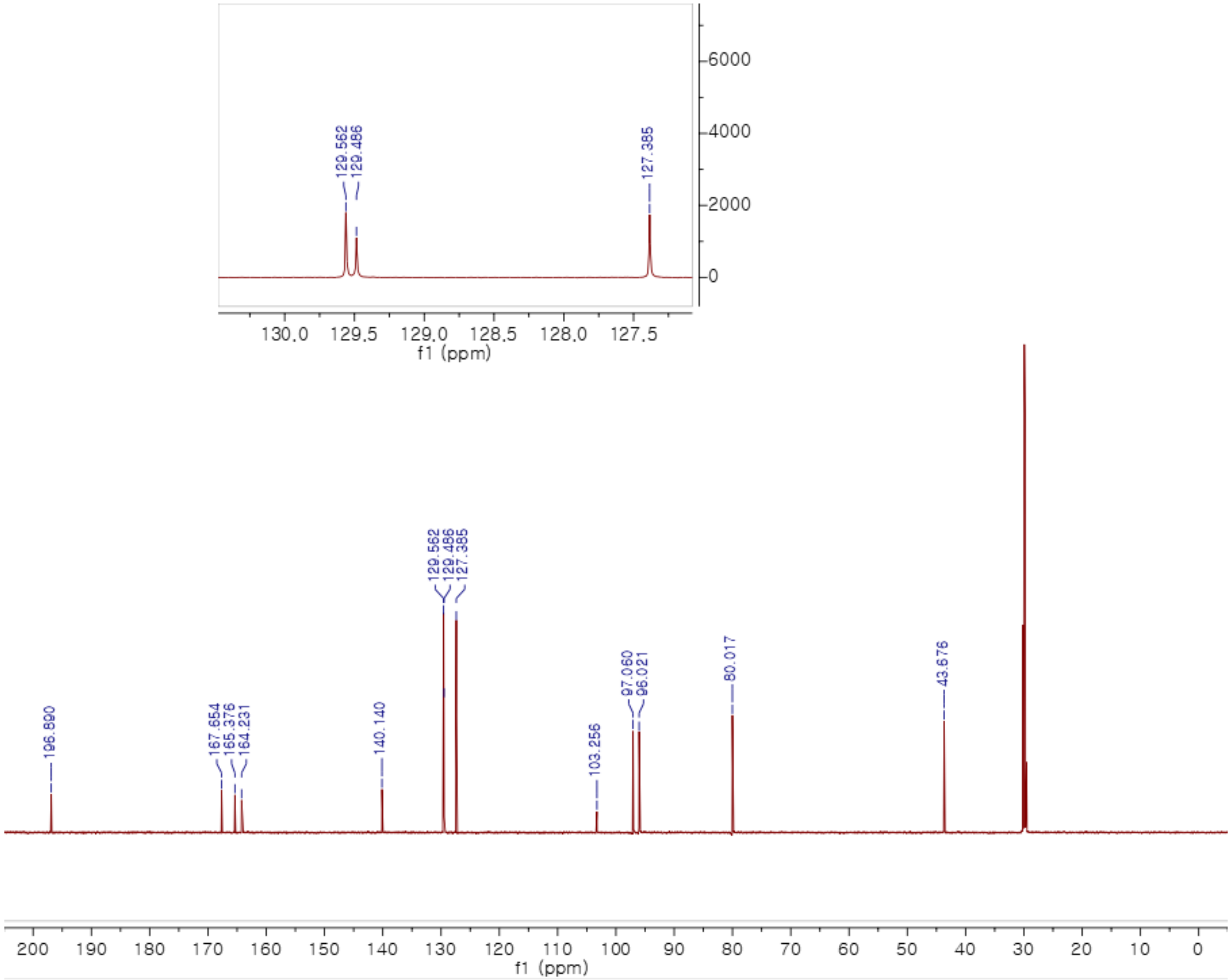
129.562
129.486
127.385
6000
4000
2000
0
130.0
129.5
129.0
128.5
128.0
127.5
f1 (ppm)
196.890
167.654
165.376
164.231
140.140
129.562
129.486
127.385
103.256
97.060
96.021
80.017
43.676
200
190
180
170
160
150
140
130
120
110
100
90
80
70
60
50
40
30
20
10
0
f1 (ppm)

Figure S5. ^{1}H and ^{13}C NMR data of cardamonin (**4**)

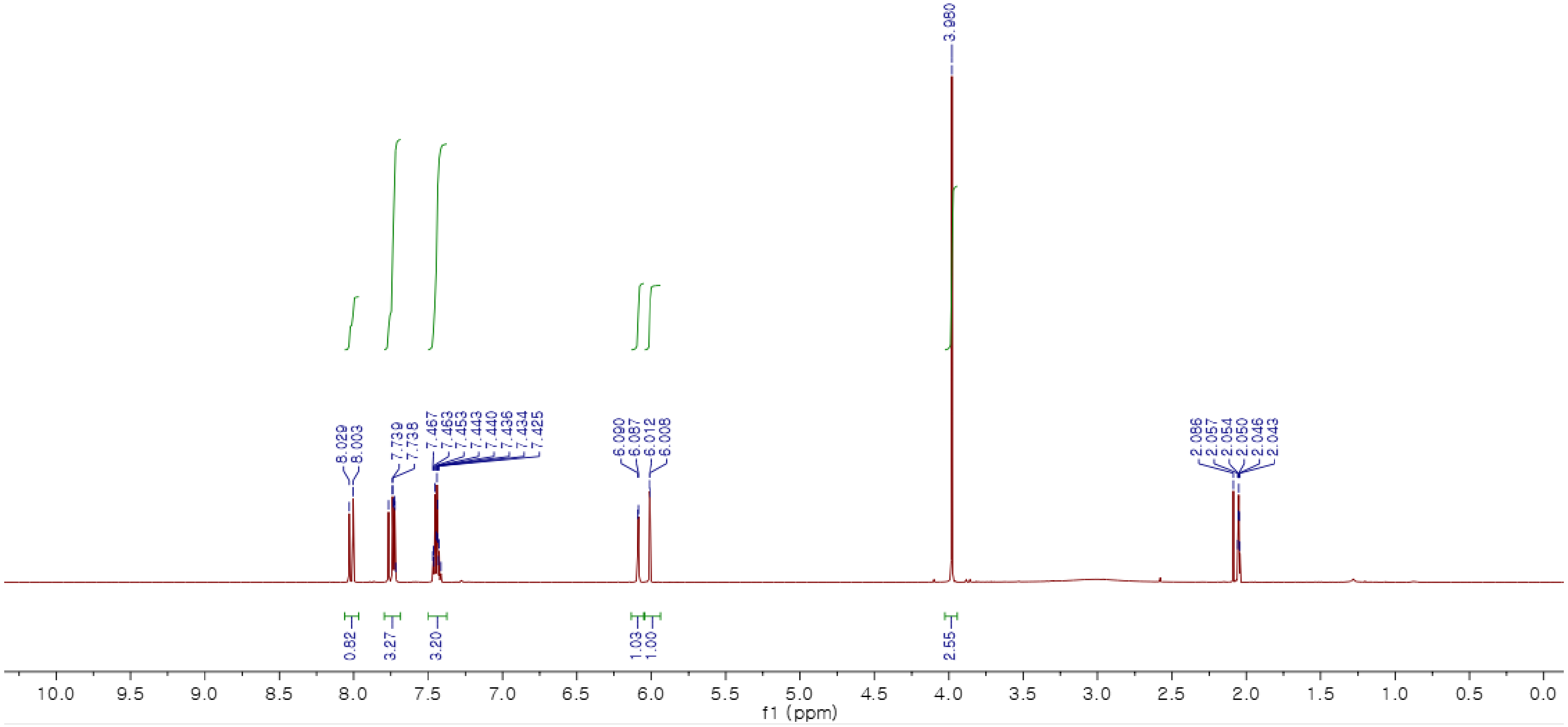

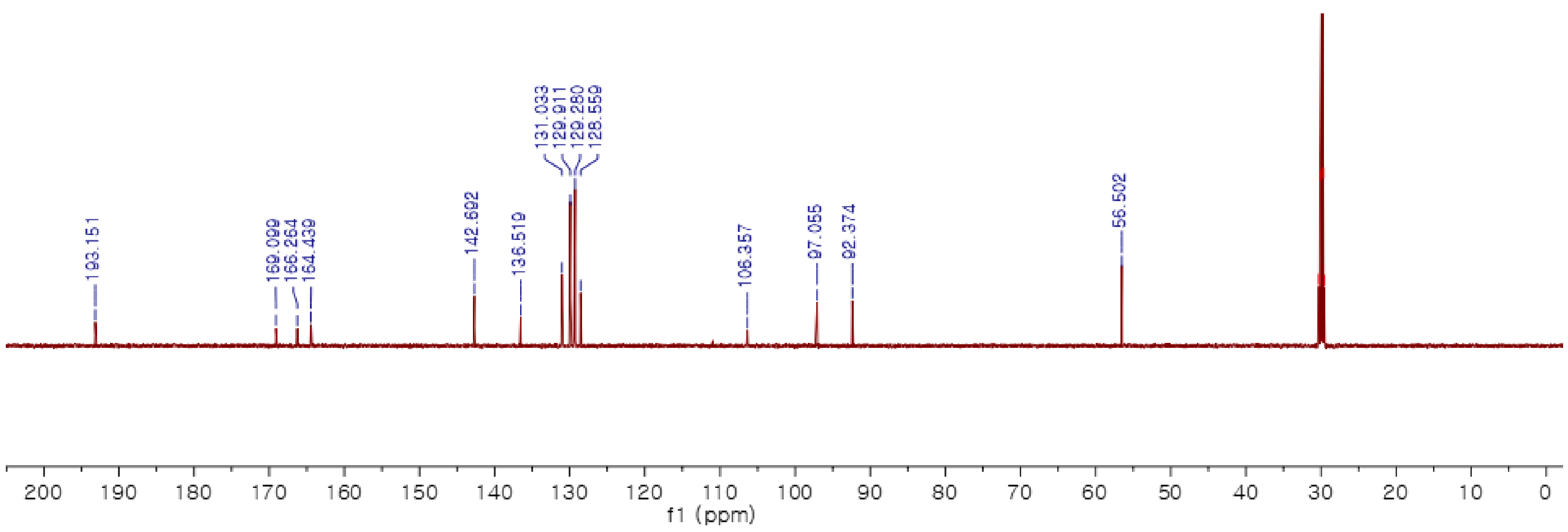
193.151
169.099
166.264
164.439
142.692
136.519
131.033
129.911
129.280
128.559
106.357
97.055
92.374
56.502
200 190 180 170 160 150 140 130 120 110 100 90 80 70 60 50 40 30 20 10 0
f1 (ppm)

Figure S6. ^{1}H and ^{13}C NMR data of pinostrobin chalcone (**5**)

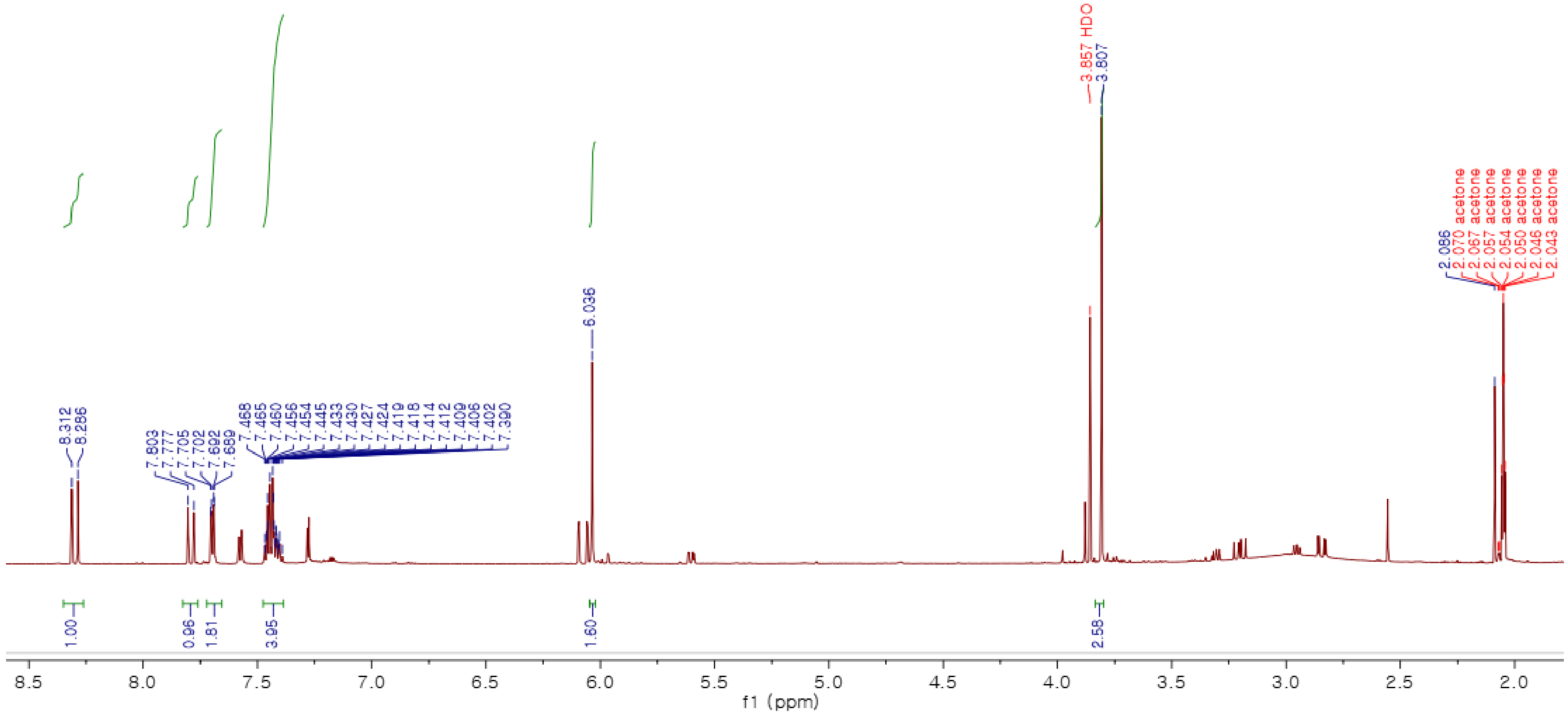

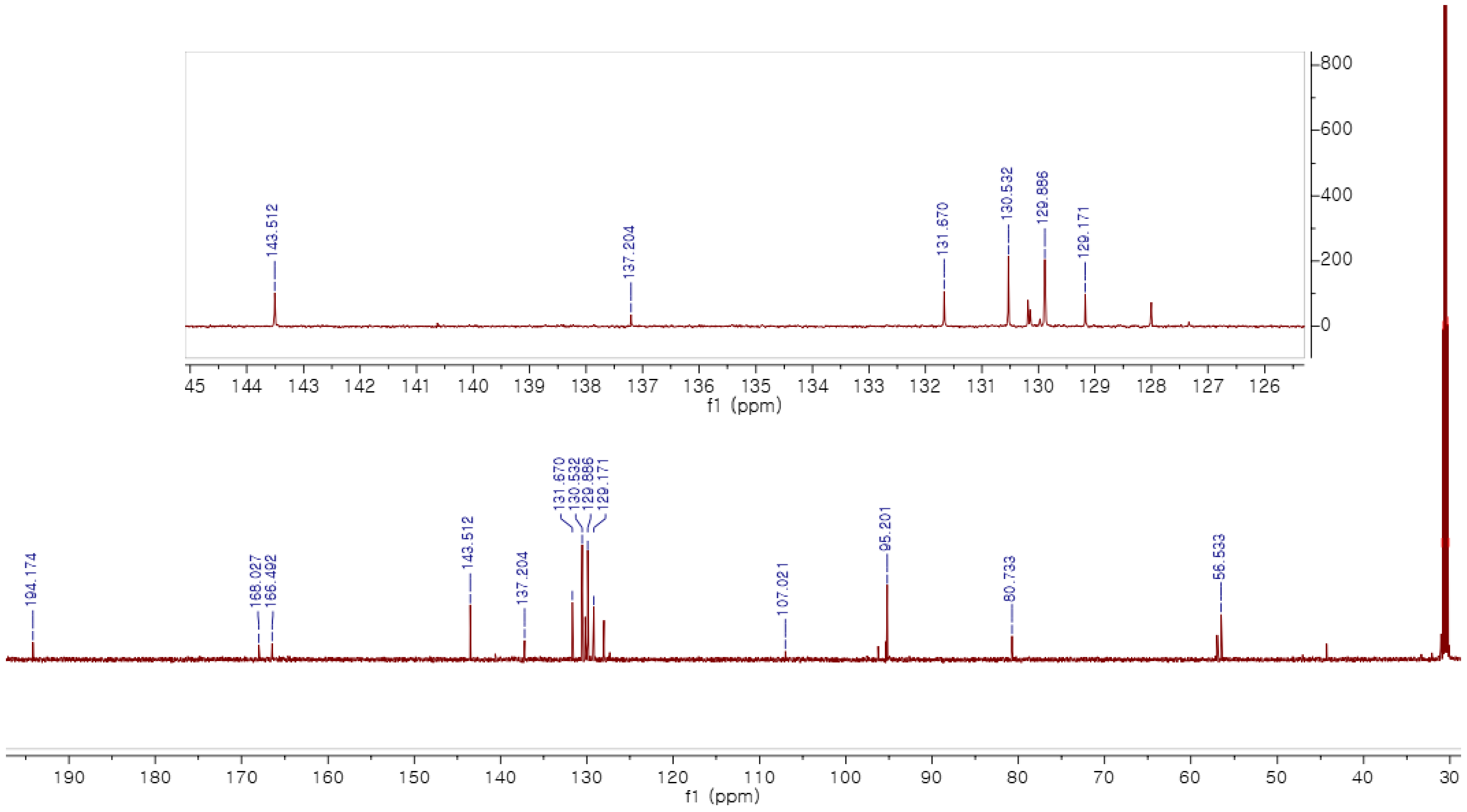
143.512
137.204
131.670
130.532
129.886
129.171
f1 (ppm)
194.174
168.027
166.492
107.021
95.201
80.733
56.533
f1 (ppm)

Figure S7. ^{1}H and ^{13}C NMR data of pinostrobin (**6**)

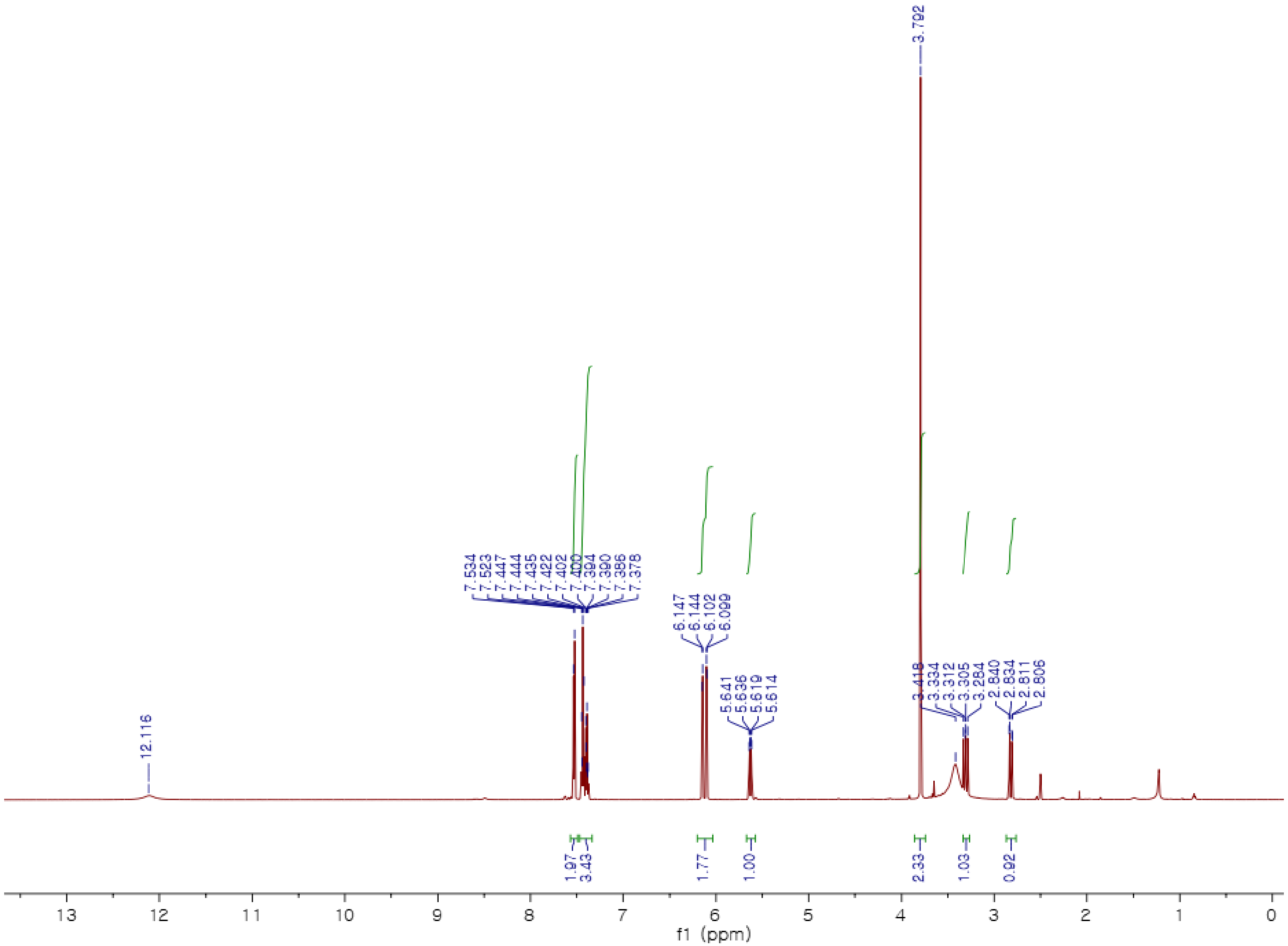

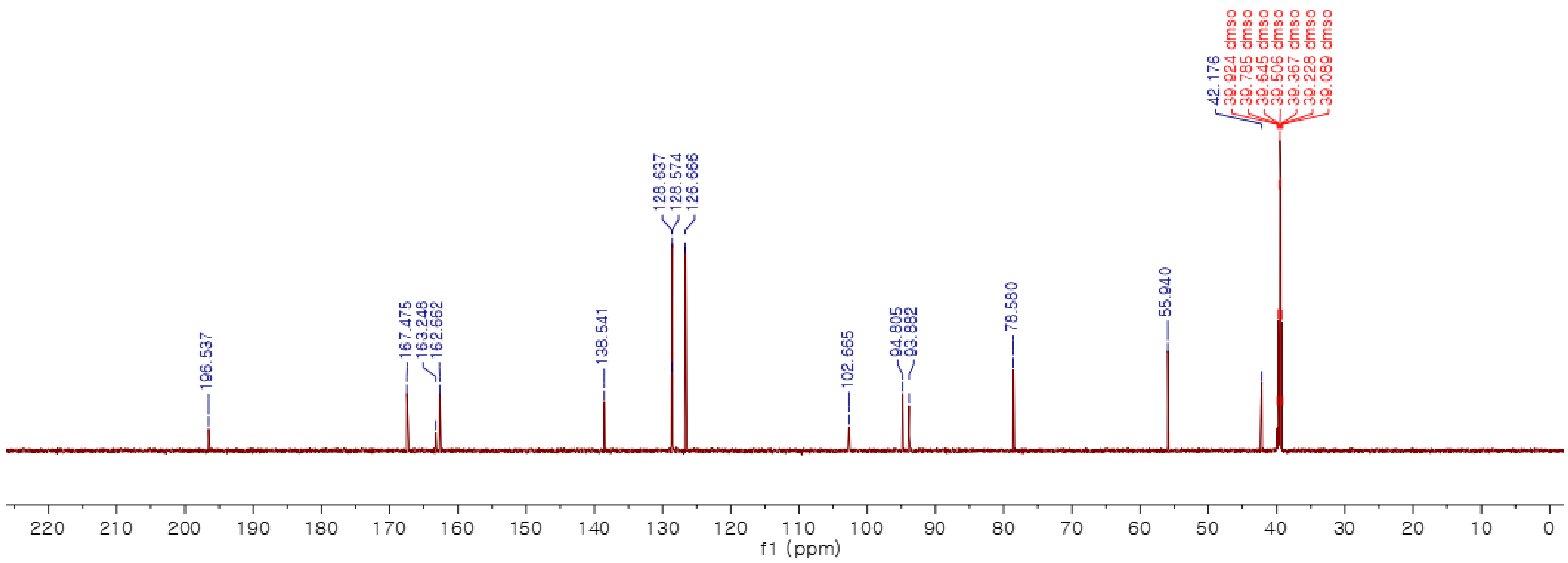

196.537
167.475
163.248
162.662
138.541
128.637
128.574
126.666
102.665
94.805
93.882
78.580
55.940
42.176
39.924 dmso
39.785 dmso
39.645 dmso
39.506 dmso
39.367 dmso
39.228 dmso
39.089 dmso
220 210 200 190 180 170 160 150 140 130 120 110 100 90 80 70 60 50 40 30 20 10 0
f1 (ppm)

Figure S8. ^{1}H and ^{13}C NMR data of 4-hydroxypanduratin A (**7**)

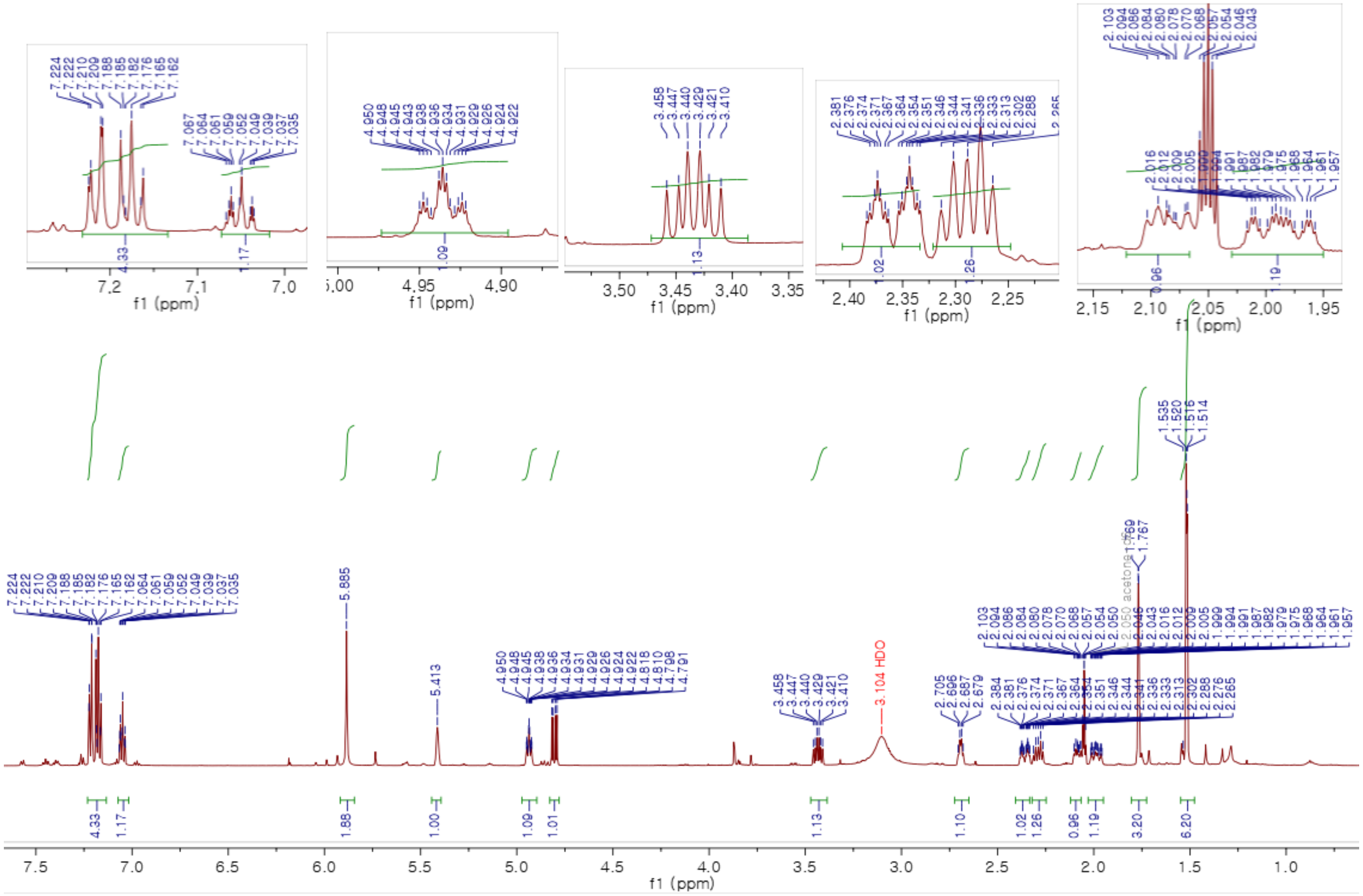

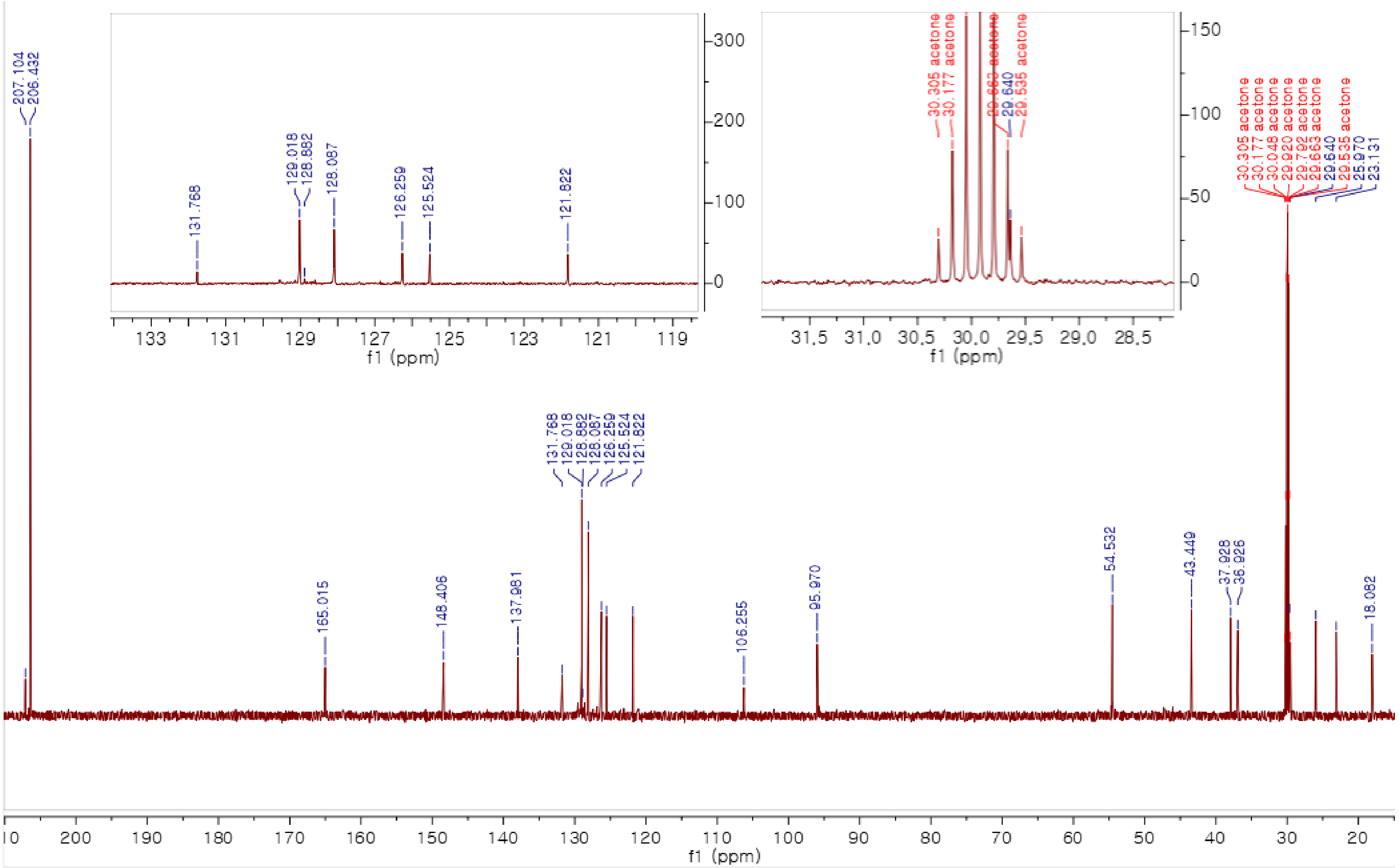
207.104
206.432
165.015
148.406
137.981
131.768
129.018
128.882
128.087
126.259
125.524
121.822
106.255
95.970
54.532
43.449
37.928
36.926
30.305 acetone
30.177 acetone
30.048 acetone
29.920 acetone
29.792 acetone
29.663 acetone
29.640
29.535 acetone
25.970
23.131
18.082
f1 (ppm)

Figure S9. ^{1}H and ^{13}C NMR data of panduratin A (**8**)

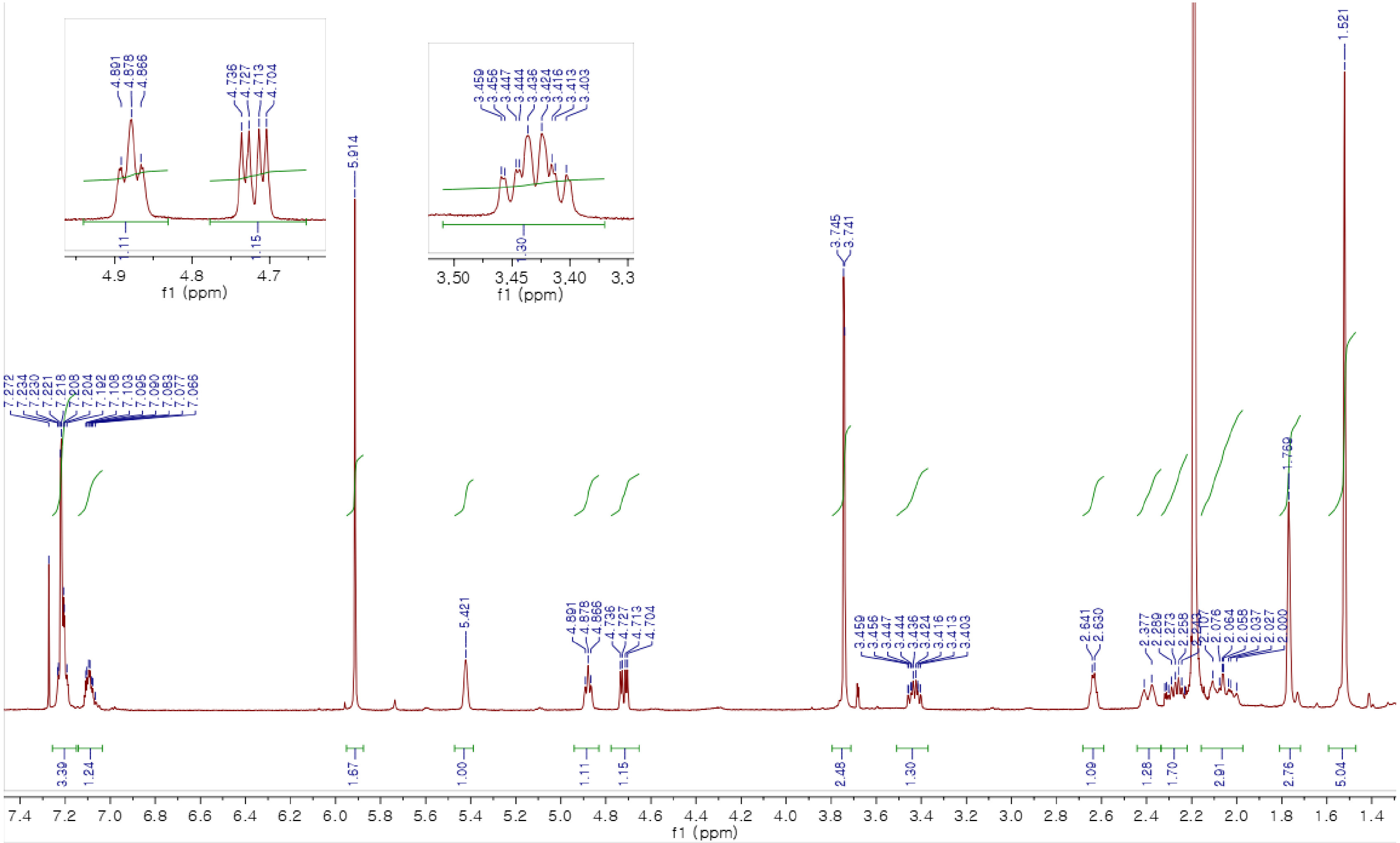

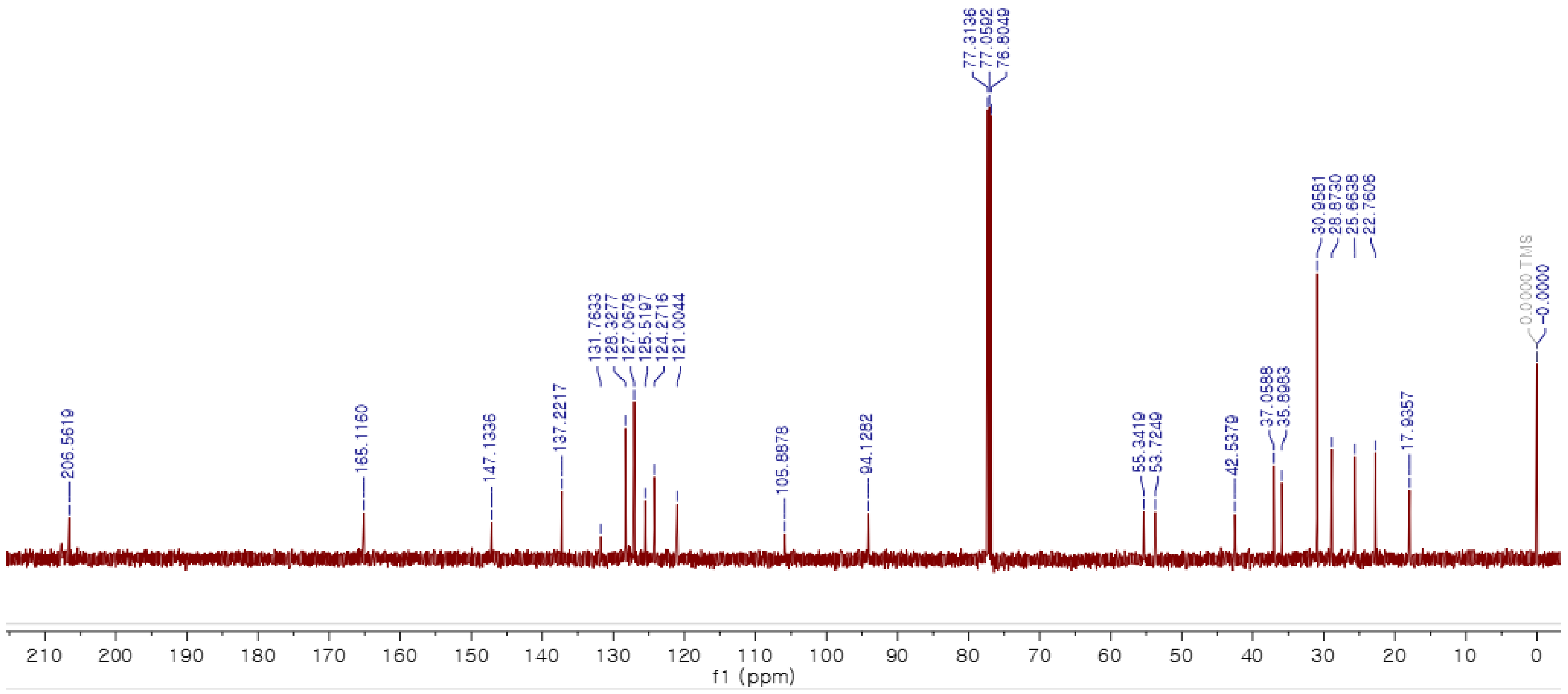
206.5619
165.1160
147.1336
137.2217
131.7633
128.3277
127.0678
125.5197
124.2716
121.0044
105.8878
94.1282
77.3136
77.0592
76.8049
55.3419
53.7249
42.5379
37.0588
35.8983
30.9581
28.8730
25.5638
22.7606
17.9357
0.0000 TMS
-0.0000
210 200 190 180 170 160 150 140 130 120 110 100 90 80 70 60 50 40 30 20 10 0
f1 (ppm)
